# Niche formation and metabolic interactions contribute to stable diversity in a spatially structured cyanobacterial community

**DOI:** 10.1093/ismejo/wraf126

**Published:** 2025-06-19

**Authors:** Sarah Jennifer Nicola Duxbury, Sebastien Raguideau, Kelsey Cremin, Luke Richards, Matej Medvecky, Jerko Rosko, Mary Coates, Kieran Randall, Jing Chen, Christopher Quince, Orkun S Soyer

**Affiliations:** School of Life Sciences, University of Warwick, Coventry CV4 7AL, United Kingdom; Organisms and Ecosystems, Gut Microbes and Health, Earlham Institute, Norwich NR4 7UZ, United Kingdom; School of Life Sciences, University of Warwick, Coventry CV4 7AL, United Kingdom; School of Life Sciences, University of Warwick, Coventry CV4 7AL, United Kingdom; Bioinformatics and Digital Health Services, Research Technology Platforms, University of Warwick, Coventry CV4 7AL, United Kingdom; School of Life Sciences, University of Warwick, Coventry CV4 7AL, United Kingdom; School of Life Sciences, University of Warwick, Coventry CV4 7AL, United Kingdom; School of Life Sciences, University of Warwick, Coventry CV4 7AL, United Kingdom; School of Life Sciences, University of Warwick, Coventry CV4 7AL, United Kingdom; Organisms and Ecosystems, Gut Microbes and Health, Earlham Institute, Norwich NR4 7UZ, United Kingdom; Quadram Institute Bioscience, Norwich Research Park, Norwich NR4 7UQ, United Kingdom; Warwick Medical School, University of Warwick, Coventry CV4 7AL, United Kingdom; School of Life Sciences, University of Warwick, Coventry CV4 7AL, United Kingdom

**Keywords:** cyanobacteria, photogranules, microbial communities, community stability, freshwater ecosystems, spatial organisation, microbial mats, metabolic interactions, carbon sharing, vitamin exchange

## Abstract

Understanding how microbial communities maintain stable compositional diversity is a key question in microbial ecology. Studies from pairwise interactions and synthetic communities indicate that metabolic interactions and spatial organisation can influence diversity, but the relevance of these factors in more complex communities is unclear. Here we used a cyanobacterial enrichment community that consistently forms millimetre-scale granular structures, to investigate compositional diversity and its stability. Over a year of passaging in media without significant carbon source, we found stable co-existence of 17 species belonging to diverse bacterial phyla. Metagenomic analysis revealed polysaccharide breakdown genes and complementary vitamin biosynthesis pathways in these species. Supporting these findings, we show growth of several isolated species on cyanobacterial slime components and experimentally verify vitamin exchanges between two members of the community. Several species had genes for (an)oxygenic photosynthesis and sulfur cycling, the expression of which we verified via metatranscriptomics. Consistent with this, we found that the granular structures displayed oxygen gradients with anoxic interiors. Cyanobacteria and other bacteria were distributed on the periphery and insides of these structures, respectively. Perturbation of the community via glucose addition resulted in fold increases of the heterotrophs, whereas disturbing the community by continual shaking led to fold reductions in several heterotrophs, including anoxygenic phototrophs. In contrast, removal of vitamins supplementation did not consistently alter species coverages, due to predicted vitamin sharing amongst community members. Taken together, these findings indicate that spatial organisation, microenvironment niche formation and metabolic interactions contribute to community compositional diversity and stability.

## Graphical Abstract

**Graphical Abstract:**
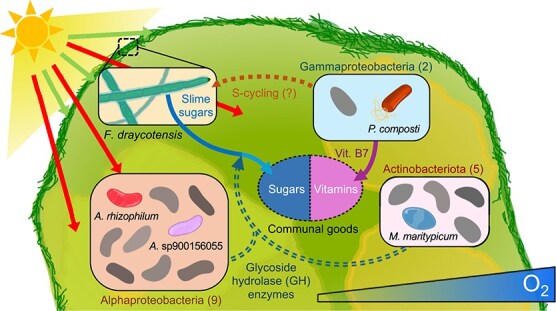

## Introduction

Understanding the assembly and stability of species composition in microbial communities is key for predicting community function. Experimental studies involving enrichment of environmental samples in defined media indicate that assembled community composition and diversity in terms of higher-level taxonomy, can be related to nutritional richness of the environment [1, 2]. For the experimental assembly of relatively low diversity microbiomes, the extent and maintenance of microbial diversity is suggested to relate to metabolic cross-feeding, and the production of secondary metabolites, for growth in minimal medium supplemented with carbon sources [2–5]. Full genomic annotations and experimental verification of species-specific metabolite exchanges, however, remain limited.

Another factor that can influence diversity and stability in a community is spatial organisation [6, 7]. Certain natural spatially organized microbial communities are indicated to maintain taxonomic stability over time, in both environmental and host-associated contexts [7–9]. Metabolic gradients found within some of these communities [10–14] are hypothesized to be important for microbial interactions and community composition [12, 15]. In a laboratory-based single species culture, spatial stratification of the environment allowed emergence of different strains maintained through competitive trade-offs (negative frequency-dependent selection) [16].

Despite these indicative results, the emergence of spatial organisation and metabolic interactions in a microbial community, and their contribution to determining species composition and stability, remain to be systematically studied. Current model systems often lack spatial structure and multi-species complexity, contrasting with natural communities that are shown to display microenvironments and high diversity [9, 17, 18]. Studies on spatial systems have focused mainly on biofilms composed of one or two species [19–21]. Thus, temporal stability of final community composition in presence of spatial organisation remains under-studied. Mechanistic studies of metabolic interactions usually involve “bottom-up” approaches [3, 22–24], using culturable microbial pairs or specific microbial groups to predict interactions and stability at the community level. It is unclear to what extent the outcomes of such pairwise interactions, in homogenous environments, will be predictive for spatially organized natural communities.

In order to study the maintenance of microbial diversity in a spatially organized system, we established here a cyanobacterium-dominated enrichment microbial community and investigated temporal compositional stability and metabolic interactions within it. Over 1 year of serial passaging in media without any significant carbon source, we found that this community consistently forms mm-scale granular structures and stably maintains co-existence of 17 phylogenetically diverse species, resolved through temporal short- and long-read metagenomics. Community members’ genomes encoded distinct metabolic functions of oxygenic and anoxygenic photosynthesis, specific polysaccharide degradation capabilities, sulfur cycling, and vitamin prototrophy or auxotrophy. In line with these results, we experimentally measured growth of isolated species on monosaccharide components of cyanobacterial-secreted polysaccharide slime, and vitamin sharing between a prototrophic and an auxotrophic species. Using meta transcriptomics, we verified expression of genes with anaerobic functions, and in conjunction, found the spatial structures formed by the community to display oxygen gradients and anoxic interiors. When we disturbed the community by breaking structural organisation or by glucose addition, heterotroph coverages showed fold decreases or increases respectively, indicating de-stabilisation. Alternatively, vitamins removal did not lead to large changes in species coverages. Taken together, these findings indicate that a photosynthetic community can develop into a spatially organised microcosm where spatial niches support metabolic functions and interactions, thereby contributing to compositional stability and diversity.

## Materials and methods

For a detailed description of all experimental methods, see *Supplementary Information* (*SI*).

### Sample collection and culture maintenance

**Figure 1:**
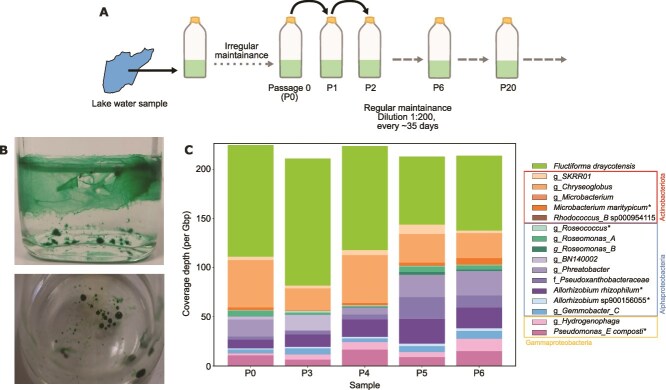
Culturing schematic, structure formation and taxonomic placement of granule-forming freshwater-derived phototrophic communities. (A) Schematic showing culturing regime from a natural, lake sample, with initial irregular culture maintenance followed by a regular laboratory serial passaging regime in culture bottles. A single culture was initiated, and several serial passages of this culture were sequenced for microbial community composition (see *Methods* for full details). (B) Representative images of the structured community. Granules of various sizes occur (*bottom*) and filamentous bundles and biofilms attaching to the base and walls of the glass bottle (*top*). (C) Community composition, i.e. species coverage, over sub-culture passages (samples) (legend and Table 1) for 17 species characterised from short-read Illumina and long-read PacBio sequencing (see *methods*). The highest taxonomic resolution is described in the legend on the figure: f = family; g = genus. Higher taxonomic order grouping is also indicated with coloured boxes and labels on the right of the legend. Asterisks next to species names indicate species that were experimentally isolated. Passage (P) number is labelled on the x-axis, denoting different samples. Data from P1 and other cultures are presented in Fig. S1.

Freshwater samples were collected from Draycote Water Reservoir, Warwickshire, UK. Samples were initially maintained through irregular sub-culturing on liquid or agar media, using minimal media without carbon source, described for cyanobacterial culturing (see *SI*). A single culture was then established for regular serial sub-culturing in BG11+ medium (DSMZ medium reference number 1593), with addition of a vitamin mix (Table S1) but without carbon source addition. For the sub-culturing regime, where sub-culturing was done approximately monthly, cultures were grown under continuous 12 h/12 h light/dark cycles with white, fluorescent illumination of 14–20 μmol photons m^−2^ s^−1^ at room temperature under static conditions. For each passaging step, we performed a 1:200 dilution by transferring 150 μl of re-suspended filamentous culture into a final volume of 30 ml of BG11+ vitamin mix (Fig. 1A).

### DNA extraction and sequencing of community samples

For shotgun sequencing*,* DNA was extracted using the Qiagen PowerSoil Pro kit (Hilden, Germany, Cat. No. 47014). DNA was sent to Novogene (Cambridge, UK) for paired-end sequencing on a NovaSeq system (Illumina). For PacBio HiFi sequencing, DNA extractions were performed by the Natural Environment Research Council (NERC) Environmental Omics Facility (NEOF) using the Macherey-Nagel NuceloBond HMW DNA kit. PacBio DNA libraries and sequencing were completed at the Centre for Genomics Research. Sequencing was performed on the Sequel II SMRT Cell in Circular Consensus Sequencing mode.

### Sequence assembly, binning, and coverage analysis

Samples from Passage (P)0 and 3–6 were processed using the STRONG pipeline (available at https://github.com/chrisquince/STRONG) and to generate metagenomically assembled genomes (MAGs) [25] (see further details in the *SI*). The PacBio HiFi samples were assembled using hifiasm-meta [26], and the resulting unitig assembly graphs were used in the downstream analyses (see further details in the *SI*). We taxonomically classified MAGs with GTDB-Tk v2.1.0 [27] and data version r207, using standard settings on genome taxonomy database (GTDB)-Tk [28]. Coverages of the MAGs in community samples were calculated by mapping sample reads onto the originally identified MAGs using bwa-mem [29], and coverage was obtained using samtools (1.17) [30] and bedtools (v2.25.0) [31]. In brief, for each MAG, we first calculated the total length of reads mapping to that MAG normalised by that MAG’s genome length. This value is then further normalised (divided) by the length of all reads (i.e. total DNA) in each sample and then multiplied by 10^9^ to be expressed in Giga base pairs. The resulting value will be proportional to the relative genome copy number in the community and hence, is a good proxy for relative abundance. Mathematically, “Coverage depth (per Gbp)” for a given MAG is given by:


\begin{align*} &\mathrm{Coverage}\ \mathrm{depth}\ \left(\mathrm{per}\ \mathrm{Gbp}\right)\\&=\frac{{Total\ length\ of\ reads\ mapping\ to\ MAG}\!\left/ \!{MAG\ genome\ length}\right.}{Total\ length\ of\ all\ reads\ in\ sample}\bullet{10}^{9}. \end{align*}

### Genome annotations

Genome annotations were performed using the DFAST annotation platform (releases 1.2.15/1.2.18; [32]). Resulting protein sequences were annotated for KEGG orthologs (KOs) using KofamKOALA (release 102.0/103.0; [33]). Resulting KO lists from short and long-read data were concatenated to create a unique KO list (where possible) for each of the MAGs/bins identified from the PacBio data. MetQy package (version 1.1.0) [34] was used to analyse the completeness of KEGG modules as defined by KEGG orthologs (KOs) in the KEGG database [35]. In addition, specific KOs for monosaccharide degradation, vitamin biosynthesis, and sulfur metabolism were searched individually across genomes. Transporters defined by KOs were also searched. Monosaccharide and vitamin pathways, and transport functions were additionally searched on the MetaCyc database [36]. Polysaccharide degradation genes were identified using the dbCAN3 database using all Markov models implemented [37]. Enzyme substrates were assigned to each dbCAN family using the dbCAN_sub database [38], either using the curated mapping table or via sub-family searches for ambiguous annotations.

### Species isolation and physiological assays

Isolation of species was achieved using three types of agar with carbon source supplementation as described in the *SI*. To profile growth of isolates, the Biolog “PM1 96 Carbon Utilisation Assay” (Biolog, Hayward, CA, USA) was used with growth measured via absorbance (optical density at 600 nm wavelength) at 30°C for 48 hours in a plate reader (CLARIOstar, BMG LABTECH GmbH, Ortenberg, Germany).

In addition, *Pseudomonas composti* and *Allorhizobium rhizophilum* isolates were growth-profiled in BG11+ medium in the presence and absence of vitamin mix over replicate growth wells in the plate reader. *A. rhizophilum* was also profiled in BG11+ medium in the presence of only biotin supplementation (same concentration as in vitamin mix) or with supplementation of *P. composti* supernatant from a culture grown in BG11+ medium without vitamin mix. Supernatant and BG11+ medium were mixed in a 20:80 ratio.

### Slime extraction and gas chromatography–mass spectrometry characterisation

Cyanobacterial slime extraction was performed adapting methods previously described [39, 40]. The final protocol used is available via protocols.io [41]. A 1.2 l culture of the community equivalent to Passage 16 was grown for three months for sufficient slime production, producing approximately 50 mg of lyophilized slime, which was analysed via GC–MS. In brief, the pellet underwent hydrolysis with sulfuric acid, and then trimethylsilylation in preparation for the GC–MS. GC–MS was performed on an Agilent 7890GC, coupled with a 5977B MSD detector. An Agilent HP-5MS with 5.0% Phenyl Methyl Silox column was used (30 m × 250 μm × 0.25 μm) (see *SI* for further details).

### Micron**-**scale probe measurement of oxygen gradients

Oxygen was measured using a Unisense OX-NP-710704 probe. See further details in the *SI*.

## Results

### A cyanobacterial enrichment culture displays reproducible spatial structure formation

**Figure 2:**
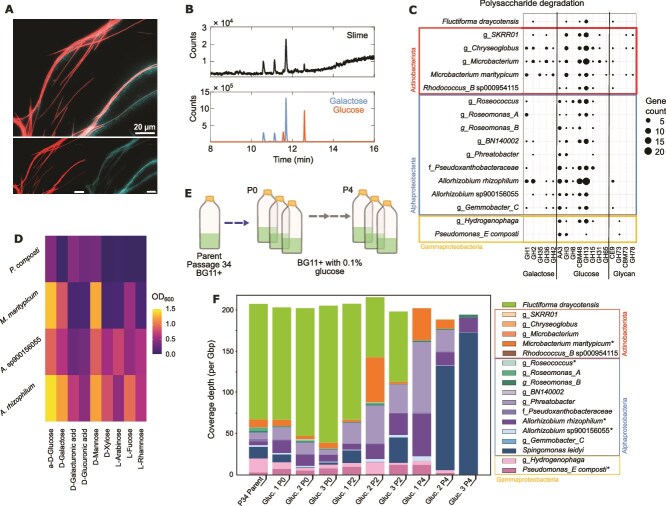
Analysis of slime-based carbon exchange in the community. (A) Attachment of bacteria to cyanobacterial slime. Shown is a microscopy image of a community sample stained with 10 μM Thioflavin T (ThT), a dead-live indicator for bacteria based on membrane potential (see *SI* for details). This fluorescent image is obtained using two different filters: ThT fluorescence imaged through a ECFP (U-F9001) filter cube, and the cyanobacterial photopigment fluorescence imaged through a HcRed (U-F41043) filter cube. Community sample was placed on a BG11+ vitamin mix medium agar pad, which was then flipped onto a cover slide (see *SI*). (B) GC chromatograms of cyanobacterial slime (exopolysaccharide), compared with chromatograms of galactose and glucose standards. The x-axis shows retention time. See Fig. S6 for mass spectra and *SI* for GC–MS analysis details. (C) Number of detected carbohydrate-active, polysaccharide degrading enzymes in community members using the dbCAN3 database (see *Methods*). The x-axis lists the different dbCAN3 families with substrate monosaccharide components indicated underneath the axis: galactose, glucose, or undefined glycans. Dot size on the plot indicates the number of genes detected in that category. The y-axis shows the identified species in the community, as listed in Fig. 1C. (D) Growth of four isolated species on carbon substrates, selected based on being commonly associated as components of cyanobacterial slime [37, 42] and based on results shown in panel (C). Endpoint optical density (OD600) following 48 hours of growth is presented across carbon sources assayed in a biolog PM1 microplate per species (see *methods*). (E) Schematic of serial passaging regime of triplicate cultures initiated from a parent culture representing a passage culture of the main community lineage. (F) Community composition over serial passaging in BG11+ vitamin mix medium with 0.1% w/v glucose addition. Species coverages are presented from short-read data for passages (P) 0, 2, and 4 from the experiment; N = 3 for each, with the same cultures tracked through time. Species taxonomy is presented in Table 1. An additional species (*Sphingomonas leidyi*) is presented that was only detected in the parent and passage cultures for this experiment (see *Results*).

We collected water samples from a local freshwater reservoir and maintained these in a minimal medium lacking any significant carbon source (see *Methods*). After an initial irregular passaging period (see *SI*), we established a regular, serial passaging regime with a 1:200 dilution approximately every 35 days (Fig. 1A). We found repeatable spatial structure formation in these cultures over passages, consisting of both spherical and more irregularly shaped granules (ranging from mm to cm scale), as well as aggregates, and connected biofilms (Fig. 1B). We refer to these structures collectively as “cyanobacterial granules”. Microscopy analyses showed these structures to be dominated by a filamentous cyanobacterium (Fig. 2A), that displays a characteristic gliding motility [42], similar to that seen in other filamentous cyanobacteria [43].

### Cyanobacterial granules harbour a diverse, mid-complexity microbial community

From five passages ((P)assage P0 and P3–6) within a regular sub-culturing regime, we extracted DNA from mature cultures, performed short-read Illumina sequencing, and co-assembled raw sequencing data (see *Methods*). The sample from P1 was collected at an earlier culture age, therefore was analysed separately (Fig. S1), whilst data were not collected for P2. Serial passaging of the community was extended to P20 and beyond, and perturbation experiments were initiated from these cultures (see below and *SI*). We also characterized community composition of a sample prior to P0 from the irregular culturing period (see *Methods* and Fig. S1). In all samples analysed, we found the same set of 16 MAGs from short-read sequence data. For each sample, above 99.0% of raw sequence data mapped to the assembly, with sequence data mapped to MAGs varying between 89.0%–99.0% across samples (see *Supplementary file 1*). To further assess the total taxonomic diversity represented by this collection of MAGs, 16S rRNA genes were annotated and grouped into operational taxonomic units (see *SI*). Each of these could be mapped to one or more MAGs. Together these analyses show that the binning effort was highly successful at characterising all the bacterial species found within this laboratory-adapted, spatially structured cyanobacterial community.

We taxonomically assigned the MAGs using the GTDB phylogenetic placement toolkit [28] (see *Methods* and Table 1). Besides the mentioned cyanobacterium, the remaining 15 species were distributed in the phyla *Actinobacteriota* (five species) and *Proteobacteria* (10 species). The latter group was spread between the *alpha*- (eight species) and *gamma-Proteobacteria* (two species, *Pseudomonas E. composti* and a species of the *Hydrogenophaga* genus). The same species were also found in P1 and P20, and in the early sample prior to P0, with a greater enrichment of the cyanobacterium in these younger culture age samples (Fig. S1). We have also noted that the system composition (in terms of species number and taxonomic identity) was robust to cryo-preservation (in 10.0% v/v glycerol), as a cryo-revived community sample (from P6) developed granules and showed comparable species composition as a similarly aged community sample prior to cryopreservation (P11) (Fig. S1).

**Table 1:** Taxonomy of bacterial species (MAGs) characterized in the structured cyanobacterial community. Assigned taxonomy of the final set of MAGs (metagenome assembled genomes) obtained from Illumina short-read and PacBio long-read shotgun metagenomics. MAGs only present in short-read sequencing are indicated by an asterisk in the “MAG” column. A MAG that was only identified in long-read sequencing is indicated by a “†”. The highest taxonomic resolution is presented for each bin. The cyanobacterial species in our system is assigned by GTDB-Tk to an automatically generated family called “JAAUUE01” (see main text for further discussion of the phylogeny of this species). We propose a new taxonomic classification for this family and species, highlighted in bold.

**MAG**	**Phylum**	**Class**	**Order**	**Family**	**Genus**	**Species**
1	*Actinobacteriota*	*Acidimicrobiia*	*Acidimicrobiales*	*Microtrichaceae*	*SKRR01*	*/*
2	*Actinobacteriota*	*Actinomycetia*	*Actinomycetales*	*Microbacteriaceae*	*Chryseoglobus*	*/*
3 *	*Actinobacteriota*	*Actinomycetia*	*Actinomycetales*	*Microbacteriaceae*	*Microbacterium*	*/*
4	*Actinobacteriota*	*Actinomycetia*	*Actinomycetales*	*Microbacteriaceae*	*Microbacterium*	*Microbacterium maritypicum*
5 *	*Actinobacteriota*	*Actinomycetia*	*Mycobacteriales*	*Mycobacteriaceae*	*Rhodococcus_B*	*Rhodococcus_B* sp000954115
6	*Cyanobacteria*	*Cyanobacteriia*	*Cyanobacteriales*	***Fluctiformaceae***	***Fluctiforma***	***Fluctiforma draycotensis* gen. nov., sp. nov.**
7	*Proteobacteria*	*Alphaproteobacteria*	*Acetobacterales*	*Acetobacteraceae*	*Roseococcus*	*/*
8	*Proteobacteria*	*Alphaproteobacteria*	*Acetobacterales*	*Acetobacteraceae*	*Roseomonas_A*	*/*
9	*Proteobacteria*	*Alphaproteobacteria*	*Acetobacterales*	*Acetobacteraceae*	*Roseomonas_B*	*/*
10	*Proteobacteria*	*Alphaproteobacteria*	*Rhizobiales*	*Beijerinckiaceae*	*BN140002*	*/*
11	*Proteobacteria*	*Alphaproteobacteria*	*Rhizobiales*	*Phreatobacteraceae*	*Phreatobacter*	*/*
12	*Proteobacteria*	*Alphaproteobacteria*	*Rhizobiales*	*Pseudoxanthobacteraceae*	*/*	*/*
13	*Proteobacteria*	*Alphaproteobacteria*	*Rhizobiales*	*Rhizobiaceae*	*Allorhizobium*	*Allorhizobium rhizophilum*
14 **†**	*Proteobacteria*	*Alphaproteobacteria*	*Rhizobiales*	*Rhizobiaceae*	*Allorhizobium*	*Allorhizobium* sp900156055
15	*Proteobacteria*	*Alphaproteobacteria*	*Rhodobacterales*	*Rhodobacteraceae*	*Gemmobacter_C*	*/*
16	*Proteobacteria*	*Gammaproteobacteria*	*Burkholderiales*	*Burkholderiaceae*	*Hydrogenophaga*	*/*
17	*Proteobacteria*	*Gammaproteobacteria*	*Pseudomonadales*	*Pseudomonadaceae*	*Pseudomonas_E*	*Pseudomonas_E composti*

To improve characterisation of community members, we performed long-read PacBio Hifi sequencing of two community samples (P0 and P7) (see *Methods*). This resulted in a total of 16 MAGs, of which 14 were common with the short-read approach (Tables 1 and S2). The two MAGs missing in the long-read analysis were the species from the *Microbacterium* genus and *Rhodococcus B* sp000954115*,* which showed decreasing coverages in the short-read data (Fig. 1C). The additional MAGs detected with PacBio represent an additional species from the *Allorhizobium* genus and an additional strain of the *Allorhizobium rhizophilum* species. Of the 16 genomes resolved by PacBio, 10 showed 100.0% completeness based on presence of a set of single copy genes (see *Methods*), whereas the remaining had completeness at 75.0% or above (Table S2). Combining short-read and long-read analyses, we resolve a final set of 17 species from this community (Table 1).

Most MAGs were taxonomically close to either a cultured species or to an uncultured MAG found on GTDB, with species-level novelty (see Table 1). The cyanobacterium had only one close homolog on GTDB, a single MAG obtained from sampling of extant stromatolites [44]. Further analysis of the phylogeny of these two cyanobacteria (see *SI*) showed that they form a family level clade within the *Cyanobacteriales* order (Fig. S2). Based on its geographical origin and characteristic motility, that sometimes lead to wave-like patterns formed by many filaments, we propose the name *Fluctiforma draycotensis* gen. nov., sp. nov. for the species found in our system and suggest a family name of *Fluctiformaceae*.

### Cyanobacterial granule community has stable species and strain composition

As a proxy for assessing stability of the community, we analysed coverage of each MAG across passages P0 and P3–6, normalised per Giga-base pair (Gbp) of sequencing to avoid biases from differences in sample sizes (see *Methods*). This showed a stable community composition over time with Shannon indices for passage cultures as 1.57 (P0), 1.40 (P3), 1.61 (P4), 2.06 (P5), and 2.00 (P6) (Fig. 1C). We have also mapped raw reads from many sub-cultured samples, as well an early sample, from the irregular culturing period, and found the same species richness and taxonomic diversity with some differences in relative coverages with culture age at time of DNA harvest (Fig. S1). At the species level, some of the coverages showed correlations with passage number, but these were not statistically significant (*P* value >0.05; Pearson’s Correlation with Benjamini-Hochberg correction) (Table S3). *F. draycotensis* was found in higher abundance than all other bins across P0 and P3–6. Using STRONG [25], a method for strain inference from longitudinal datasets, we identified two strains within each of the three species assigned to the *Allorhizobium* and *Chryseoglobus* genera, and the *Pseudoxanthobacteraceae* family (Table 1). All six strains were detected in all analysed passage samples, but for each species, one strain was dominant (Fig. S3).

Taken together, these results show a stable overall composition within the granule community over a one-year period, both in terms of species richness and taxonomic identity, and co-existence at both species and strain levels.

### Carbon fixation and slime-based carbon provision in the cyanobacterial community are predicted by genomic analyses and supported by isolate- and community-based experiments

The stable species co-existence in this spatially structured cyanobacterial community, together with the fact that our culture media lacked any significant carbon source, implies the presence of carbon sharing among community members. To explore this hypothesis, we used the MAGs from both short- and long-read sequencing and analysed metabolic capabilities (see *Methods*). As expected, the cyanobacterium *F. draycotensis* has the genes for oxygenic photosystems I and II, and the Rubisco genes *rbcL* and *rbcS* (*Supplementary file 2**)*. The species from the *Pseudoxanthobacteraceae* family and the *Hydrogenophaga* genus, also have both Rubisco genes. These results suggest that carbon provision by some of these species supports growth of the other species in the community.

A possible source of carbon is secreted “slime” (exopolysaccharide) from *F. draycotensis* that displays gliding motility. Slime is essential for gliding motility and forms an external trail around cyanobacteria [42]. In microscopy analyses, we regularly found these slime trails to be colonized by bacteria (Fig. 2A). Slime is shown to have a diverse composition of monosaccharides including galacturonate and galactose [39]. In line with these previous findings, we found that the slime in our system contains galactose and glucose among its monosaccharide components (see *Methods*) (Fig. 2B and Fig. S4).

We have found that all species in the community possess genes encoding carbohydrate degradation enzymes, particularly glycoside hydrolases (GH families) (Fig. S5) including those with substrate specificities for glucose, galactose, fucose, mannose, xylose, arabinose, and rhamnose, which are described as key components of cyanobacterial slime [39, 40, 45]. Genes with specificity for galactose and closely related substrates (within dbCAN families GH1, 2, 35, 36, and 42 – see *Supplementary file 3*) are found in several species, with *Microbacterium maritypicum* and *A. rhizophilum* displaying high presence of such genes (Fig. 2C). We have found that these genomically identified genes classified as galactose specific, are expressed in mature cultures (Fig. S6 and *SI Methods*). Additionally, presence of genes for monosaccharides transport and degradation was identified across species, particularly in *A. rhizophilum* (Fig. S7), suggesting use of monosaccharides for growth of community members.

To further support the hypothesis of slime-based carbon exchange, we attempted isolation of individual species from the cyanobacterial community to test growth on these and other carbon sources*.* Via dilution plating (see *Methods)*, we were able to isolate *P. composti*, *A. rhizophilum*, *A. sp900156055,* and *M. maritypicum*. We performed Biolog growth assays with these isolates (see *Methods*) and observed growth on galacturonate and glucuronate for *A. rhizophilum, A. sp900156055,* and *P. composti* (Fig. 2D). In addition, growth, particularly for *A. rhizophilum, A. sp900156055,* and *M. maritypicum*, was seen on several other sugars (glucose, xylose, mannose, fucose, arabinose), in line with the above summarized gene presence.

In the absence of external carbon, slime-mediated and other carbon exchanges are expected to contribute to community composition and its stability. This leads to the hypothesis that provision of external carbon sources should disrupt the system stability and result in increased heterotrophic species abundances. These heterotrophs are predicted to be growth-limited in the system by slower release of carbon from cyanobacterial sources. To test this hypothesis, we set up three replicate cultures from the main culture lineage (P34) and sub-cultured these for four passages in media with added glucose (see Fig. 2E and *Methods*). We detected an additional species (*Sphingomonas leidyi)* in P34 (see Fig. 2). It is likely that this species entered the system as a contaminant between P34 and P20 (as it was not detected in P20 or earlier ones, see Fig. S1).

Across the four sub-culturing steps with glucose addition, we found a significant difference in community composition across sub-cultures P0, P2, and P4 with glucose addition (PERMANOVA (Adonis test) with 999 permutations: Bray-Curtis stress = 8.61e-05, *F*_2,6_ = 4.67, *R*^2^ = 0.61, *P* value = 0.006) (Fig. 2F). Despite species-specific non-significant differences in coverages (Kruskal-Wallis tests with Benjamini-Hochberg correction for multiple comparisons), large fold-changes occurred. Median coverage (per Gbp) of *F. draycotensis* showed a decreasing trend and reduced by 5434.3-fold between P0 and P4 (Fig. S8). Heterotrophs of the *Alphaproteobacteria* showed an increasing trend over passages. Comparing between P0 and P4, fold increases in median coverage (per Gbp) occurred for the species from the *Phreatobacter* genus (1.9-fold), *A. rhizophilum* (1.3-fold), species from the *Gemmobacter_C* genus (1.7-fold), and *S. leidyi* (48.4-fold).

### Vitamin exchanges are predicted from genomic data and supported by isolate- and community-based experiments

**Figure 3:**
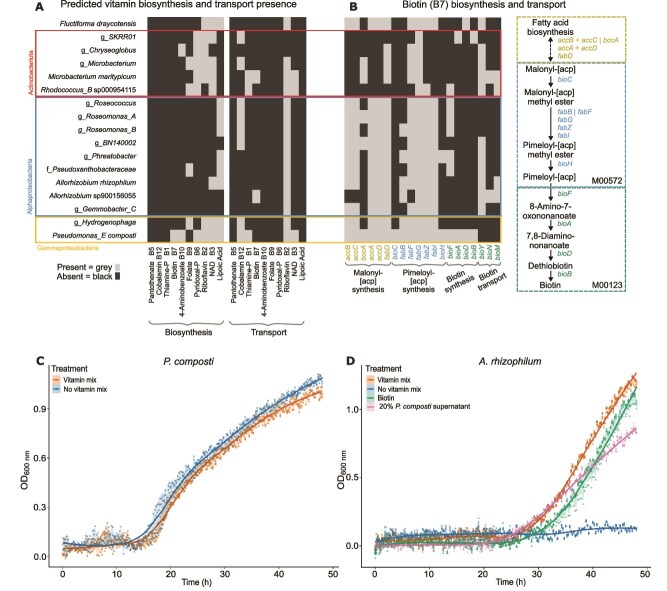
Genome-based analysis of vitamin biosynthesis pathways and experimental verification of biotin (vitamin B7) exchange. (A) Presence/absence of vitamin biosynthesis pathways and transporters, as defined by KOs (KEGG Orthologs) and MetaCyc databases. Presence (light grey) and absence (dark grey) of select functions are shown on the x-axis, across community members. See Table 1 for full species names and taxonomy, and *methods* in *SI,* and *supplementary file 4* for a full description of pathways and genes analysed. (B) Presence/absence of biotin (vitamin B7) pathway genes, as defined by KOs. Heatmap plot shows presence (light grey) and absence (dark grey) of select genes, shown on the x-axis, across community members. See *methods* for full details and *supplementary file* 4 for gene annotation and analysis. Defined pathway steps and genes required for full synthesis of biotin are illustrated on the pathway schematic, vertical bars separate alternative genes and plus symbols show required gene combinations. (C) Growth of *P. composti* in BG11+ vitamin mix medium supplemented with 0.8% w/v glucose, in the absence and presence of vitamin mix. N = 3 per treatment. (D) Growth of *A. rhizophilum* in BG11+ medium supplemented with 0.8% w/v glucose, in the absence and presence of vitamin mix or with separate supplementation of biotin, or *P. composti* supernatant mixed with BG11+ medium in a 20:80 volumetric ratio (1× BG11+ medium final concentration). N = 4 per treatment. Each treatment is represented by a different colour and assigned data-point symbol, where each datum point is the mean across the replicates. The shading shows the standard deviation between the replicates at each time point, with a smoothed line used to show the growth trends for each treatment series.

Other possible contributors to stable species co-existence are amino acid or vitamins exchanges [46–48]. We did not find any clear trends in the variation in amino acid biosynthetic capacity across the genomes (Fig. S9). We have found, however, that vitamin biosynthetic pathways—for 10 key vitamins that are commonly used in microbial growth media supplementation (Table S1)—showed varied completeness across species (Fig. 3A and *Supplementary file 4*). For individual vitamins, number of species having complete biosynthetic pathways varied from one to nine, with *P. composti* having six complete pathways (Fig. 3A). Vitamin transporters were detected for half of the vitamins, each found in at least two species. For B5 and B1, several species had near-complete pathways (B5) or patchy presence (B1) (Fig. S10). For B12*, P. composti* and *A. rhizophilum* had near-complete gene sets, whilst all other species had incomplete B12 pathways (Fig. S11). For vitamin B7, *P. composti* showed complete presence of all biosynthetic genes, whereas several genes, particularly for the lower part of the synthesis pathway, were missing across all other species (Fig. 3B and *Supplementary file 4*).

These findings suggest that *P. composti* is the species with the most complete vitamin biosynthesis pathways and may provide B7 (biotin) to other species. Indeed, we found that *P. composti* could grow to high density with or without vitamin mix addition with average endpoint growth density even slightly greater (1.07-fold) in the absence of vitamin mix (two-sample t-test: t_(4)_ = −3.33, *P* value = 0.029) (Fig. 3C). Growth of *A. rhizophilum* was greatly limited in the absence of vitamin mix, with endpoint growth density reduced by 9.6-fold (one-way ANOVA: F_(3, 12)_ = 2581, *P* value <2e-16; Tukey pairwise *P*-adj = 2.7e-14) (Fig. 3D). Supplementation of BG11+ medium containing 0.8% w/v glucose, with vitamin B7 or with supernatant collected from a grown culture of *P. composti* (see *SI)* restored growth of *A. rhizophilum* above that of the no vitamin mix treatment (Tukey pairwise adjusted *P* value = 2.7e-14; 3.7e-14 respectively) but below the level of full vitamin supplementation (adjusted *P* value = 4.2e-07; 1.4e-08 respectively) (Fig. 3D). This shows that *A. rhizophilum* is auxotrophic for vitamin B7 and provision from *P. composti* can restore growth.

Given the finding that vitamin exchanges are possible amongst community members, it is predicted that changes in vitamin composition of the culturing medium will not affect the community composition through large fold-changes in species abundances. To test this hypothesis, we set up six replicate cultures from the main culture lineage (P19) consisting of three replicates in BG11+ medium with vitamin mix and three cultures without vitamin mix addition. These cultures were serially passaged in these two medium types for 10 growth passages (see *SI*). We found sustained growth and spatial structure formation similar to the original cultures (Fig. S12). Biomass, assessed over passages one to five, significantly varied between the two treatments (medium with or without vitamin mix) when taking into account variation across passages (χ^2^_1_ = 10.287, *P* = .00134), with an average 1.6-fold reduction in biomass without vitamin mix addition (Fig. S12B). We found non-significant differences in community composition comparing the tenth passage cultures (PERMANOVA (Adonis test) with 999 permutations: Bray-Curtis stress = 0.048, *F*_1,4_ = 1.16, *R*^2^ = 0.22, *P* = .30). Supporting our predictions, differences in species coverage between treatment groups were small, when taking into account the starting composition (“parent”) per treatment (Fig. S12). Without vitamins, fold changes in median coverages of the more abundant species included a 1.3-fold decrease for *Roseomonas_A*, 1.7-fold decrease for *Hydrogenophaga*, 1.8-fold increase for *Phreatobacter*, 2-fold increase for *Pseudoxanthobacteraceae*, and 2.3-fold increase for *Pseudomonas_E composti*) (Fig. S12D). These findings indicate that vitamin exchanges within the community can sustain the species composition with little change in relative abundances.

### Spatial oxygen measurements indicate oxic/anoxic interface in granules and meta-transcriptomics shows expression of anaerobic gene functions

**Figure 4:**
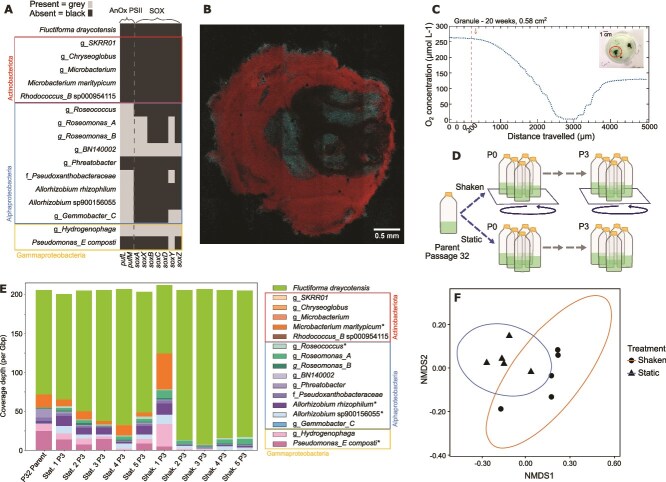
Genome-based analysis of genes with anoxygenic function and experimental verification of spatial organisation within granules with oxic/anoxic interfaces. (A) Presence/absence of anoxygenic photosynthesis and sulfur oxidation genes, as defined by KOs (KEGG Orthologs). Presence and absence of select genes are shown on the x-axis, across community members. See Table 1 for full species names and taxonomy. (B) Distribution of bacteria in granules. Shown is a tiled set of microscopy images obtained using a confocal microscope with spectral emission detection (see *SI* for details). Fluorescence excitation and emission pairings are as follows: stain Hoechst 33342- excitation: 405 nm, emission: 440–480 nm. Cyanobacteria autofluorescence- excitation: 561 nm, emission: 640–690 nm. (C) Representative oxygen profile of a cyanobacterial granule. The y-axis shows oxygen concentration, whereas the x-axis shows the distance travelled from the surface towards the interior of the granule, with 0 μm representing the starting position of the microelectrode probe at one mm above the granule. The probe is moved with a z-axis step program (200 μm step, every 20 seconds). The first descending step of the experiment and the granule prior to the oxygen profile measurement (*inset*) are shown. (D) Schematic of structure perturbation experiment through establishment of five replicate cultures under shaken and under static conditions. Each culture set was initiated from a parent culture of the main community lineage and passaged over three serial sub-cultures. (E) Community composition over serial passaging with (Shak. = shaken) and without (stat. = static) culturing perturbation. Species coverages are presented from short-read data for P3 from the experiment; N = 5 for each. Species taxonomy is presented in Table 1. (F) NMDS plot with Bray-Curtis distance to illustrate community structures under static or shaken perturbation (species abundances). N = 5 per treatment. Ellipses represent 95% confidence intervals.

Genomic annotations confirmed the photosynthetic capability of *F. draycotensis*, as expected, but also revealed anoxygenic photosynthesis capability in nine species (Fig. 4A and *Supplementary File 2*). Anoxygenic photosynthesis is an alternative type of photosynthesis whereby alternative electron donors such as hydrogen sulfide—are used, instead of water, resulting in no oxygen production [49]. Several genomes contained genes involved in assimilation and cycling of sulfur (Fig. 4A and Fig. S13). Two species, those from the *Hydrogenophaga* and BN140002, showed capability of anaerobic thiosulfate oxidation (SOX complex), whilst five species showed capability of sulfite oxidation (all species had a complete SOE complex). Several species, particularly *P. composti* and the species from the *Pseudoxanthobacteraceae* family showed capability for sulfate and sulfite reduction via the assimilatory pathway. In contrast, we did not find presence of dissimilatory sulfate reduction genes used in anaerobic respiration [50] across any of the genomes (Fig. S13). Additionally, we found that *P. composti,* the species from the *Pseudoxanthobacteraceae* family, and *F. draycotensis,* contain alkanesulfonate, thiosulphate and sulfate transport genes, and genes for subsequent conversion to sulfite and polysulfide (Fig. S13). These findings suggest interspecies sulfur exchanges via secretion and uptake, as well as the presence of a sulfur cycle in the system, through sulfate reduction to sulfite and hydrogen sulfide, and subsequent oxidation of these compounds – and related thiosulfate and sulfur [51, 52] – back to sulfate via the SOX and SOE complexes (Fig. S13).

To support genome-based findings on anaerobic functions, we performed meta transcriptomics on mature cultures (see *SI*) and found that both SOX genes from *Hydrogenophaga* and BN140002 and the anoxygenic photosynthesis genes from several alphaproteobacteria were expressed in these cultures (Fig. S14). In addition, cross-sectional imaging of granules showed cyanobacteria and other bacteria were mostly confined in the periphery and insides of the granules, respectively (Fig. 4B). Performing metagenomics on DNA extracted from insides of mature granules (see *SI*) showed increased relative coverage for bacteria other than cyanobacteria (Fig. S15). Taken together, these findings suggest that non-cyanobacterial species are located in the interior of the granule where there might be anoxic regimes. To test the latter proposition, we used micron-scale electrochemical probes to measure oxygen across cyanobacterial granules. This revealed a steep inwardly decreasing oxygen gradient across the granule with the granule core being almost fully anoxic (Fig. 4C). This oxygen gradient was measured consistently across a range of differently sized and aged granules (Fig. S16).

The formation of oxygen gradients could result in spatial organisation of species within granules, which could contribute to the stability of community composition. To test the hypothesis that spatial structure stabilizes community composition, we set up two sets of five replicate cultures from the main culture lineage (P32) and performed three sub-culturing steps under shaken or static culturing (five cultures per condition) (see *SI*). Community composition did not significantly differ between shaken and static cultures from the final passage (P3) (PERMANOVA (Adonis test) with 999 permutations: Bray-Curtis stress = 0.046, *F*_1,8_ = 0.70, *R*^2^ = 0.08, *P* = .462) (Fig. 4F). We observed decreases in median coverage (per Gbp) for 10 species, between the shaken compared with static condition and in relation to the parent culture (Fig. S17). Five of the nine species with anoxygenic photosystem II (Fig. 4A) showed a reduction under shaken conditions, supporting our predictions that shaking disrupts structure formation and presence of anoxic zones. Of the anoxygenic phototrophs, reductions in median coverage were 2.5-fold for the species from the genus *BN140002*, 4.1-fold for the species from the *Pseudoxanthobacteraceae* family, 6.6-fold for *A. rhizophilum,* 6.0-fold for *Gemmobacter_C,* and 4.9-fold for the species from the *Hydrogenophaga* genus. Across the other five species that showed coverage decreases (species from the *Chryseoglobus* and *Phreatobacter* genera, *M. maritypicum*, *Rhodococcus_B_sp000954115,* and *P. composti*), fold changes ranged from 1.4-fold for the species from the *Chryseoglobus,* to 99.1-fold for *M. maritypicum.* These findings support the hypothesis that spatial structure is a key contributor to stability of species abundances.

Per species differences in coverages were non-significant when using the Benjamini-Hochberg correction for multiple comparisons following Kruskal-Wallis tests. Part of the reason for this is that the trends in coverage described above were not observed in one of the five replicates cultured under shaken condition (replicate 1, Fig. 4E). This could indicate shaking being inefficient in breaking up the structure formation. Supporting this possibility, we find that both the replicate one sample (Fig. 4E) and “inside granule” samples (Fig. S15) show prevalence of *M. maritypicum*, *A. rhizophilum,* and the species from the *Hydrogenophaga* genus. If we remove replicate one sample as an outlier, differences in species abundances between “static” and “shaken” conditions become significant (PERMANOVA (Adonis test) with 999 permutations: Bray-Curtis stress = 0.016, *F*_1,7_ = 13.88, *R*^2^ = 0.66, *P* = .021). In this case, significant differences in nine species’ coverages, including three of the anoxygenic phototrophs, were detected between the treatments (*P* = .027 for all significant comparisons), taking into account Benjamini-Hochberg correction for multiple comparisons.

## Discussion

We have shown that a spatially structured cyanobacteria-dominated microbial community, kept in the laboratory, displays temporally stable composition of 17 species, some with strain-level diversity. Through combined genomic analyses and experimental measurements we found support for cyanobacterial slime-mediated carbon exchange between a cyanobacterium and heterotrophs in the system, and direct evidence of vitamin exchange among heterotrophs. We have also shown that the spatial structures, forming granules in repeatable fashion, result in oxygen gradients, with interiors of granules becoming anoxic. We found that the cyanobacterium occupies the periphery, and the non-cyanobacterial species occupy the interiors of these granules, along with expression of anoxygenic functions using metatranscriptomics. Disrupting these structures, via culture shaking, or providing glucose as an external carbon source, both resulted in shifts in the relative abundances of community members over sub-culturing rounds. Taken together, these findings show that metabolic exchanges, and particularly carbon provision from cyanobacteria to heterotrophs, and metabolic niche formation, through repeatable granule formation, contribute to compositional stability in this community. These findings will be relevant for the study of other, spatially organised, mid-complexity communities, whilst the presented system provides a useful experimental system where it is possible to link microbial diversity with functional traits, an area still largely under-explored for nature-derived communities [53–55].

Overall, the composition of the presented community has similarities, at higher taxonomic levels, to that found in natural samples collected at a single or few time points across cyanobacterial aggregates, blooms, and photogranules [17, 56–62]. Members of the orders *Rhodobacterales*, *Rhizobiales*, *Burkholderiales*, and *Pseudomonadales* [63, 64], and the *Hydrogeonophaga* genus [56, 63] are commonly detected in cyanobacterial blooms. Members of *Verrucomicrobiota* and *Planctomycetia* that are implied to be enriched in blooms, and photogranules [62], are missing in the presented system. One study that undertook longer lab-culturing of a cyanobacterial mat-derived samples found a cyanobacterial community of similar size and composition as presented here [17]. A core cyanobacterial microbiome has also been suggested based on sampling and culturing from several freshwater environments [65]. These were enriched for *Bacteroidetes* and *Proteobacteria* phyla, and specifically the orders *Rhizobiales* and *Rhodobacterales* [65]. These studies, including ours, indicate that there is a core cyanobacteria-associated community (i.e. a *cyanosphere*) that can be stably recovered, whereas there are also peripheral community members that may be lost over time under laboratory conditions.

Metabolic interactions likely provide a basis for symbioses, as previously implicated in aquatic environments and microbial mats [56, 66–69]. Given that we observe long-term survival and growth of community cultures over more than a year of sub-culturing in carbon-free media, we predict that metabolic exchanges are essential for the presented community and that this community may provide an example of a self-sustaining ecosystem [70]. Indeed, the provision of additional glucose in the system caused significant change in species abundances over time, with specific heterotrophs increasing and the cyanobacterium decreasing. This shows that compositional stability in the cyanobacterial community links to limited supply of carbon from the cyanobacterium in the absence of external sources.

We found that slime secretion and degradation could constitute a key route for carbon provision from cyanobacteria to heterotrophs. Carbon excretion from phototrophs has been suggested to influence heterotroph community assembly [17, 62, 71, 72] and in the case of slime-producing cyanobacteria, such influence can arise through selectivity of bacteria that can attach to or degrade specific types of slime. In line with this possibility, we found many species to harbour genes and pathways for degradation of exopolysaccharides and their component monosaccharides commonly found in slime. Selective mechanisms based on mucus are also described for microorganisms associated with coral [73] and squid [74] species and recently shown in the vertebrate gut [75].

We have identified both functional potential and experimental support for vitamin exchanges in the presented community, extending on previous predictions made for a similarly low-complexity cyanobacterial community [66] and *Microcystis* containing bloom communities [56]. The former study of a uni-cyanobacterial community used genome-based metabolic reconstruction to show variable presence of biosynthesis and salvage pathways for B-vitamins in different heterotrophs in that system [66]. These findings were supported with growth experiments of isolated species in monocultures [66], but vitamin exchanges among community members were not experimentally verified. In the presented system, we found a similar genomic variability for vitamin biosynthesis across species, in particular for vitamin B7 and B12. Using co-culture and supernatant experiments, we showed vitamin B7 exchange among specific community members. We have also shown that the presented community can maintain the same set of species in the absence of vitamins, with only small changes in species relative abundances. This finding further supported the capacity of vitamin exchange in the system. Overall, these findings align with the broader suggestion of vitamin and amino acid auxotrophies being common in microbial ecosystems [66, 69, 76–78] and the recent finding of core bacterial species associated with freshwater cyanobacteria being enriched in vitamin B12 biosynthetic genes [65].

The presented system displays consistent granule formation likely mediated by cyanobacterial motility. We have consistently quantified decreasing oxygen gradients across granules, with larger granules fully lacking oxygen at their core. We have shown that the cyanobacterium is mostly in the periphery of the granules, whereas non-cyanobacterial species are found in the oxygen-poor granule core. We have also shown genomic presence and expression of anoxygenic photosynthesis genes, as well as genes relating to sulfur cycling. The oxygen gradients found in the presented system are similar to those described in cyanobacterial mats [10, 79] and granular systems derived from bioreactors [62, 80]. It is possible that such gradients of oxygen, and other metabolites, can drive specific positioning of different species within spatially organised systems [62, 79, 80]. Indeed, disrupting structure formation in this system resulted in alterations in species abundances in a few sub-culture steps. A trend towards an increased cyanobacterial abundance and decreased abundances of species with anoxygenic functions indicates that spatial structure formation contributes to stable community composition. Further studies can probe this suggestion more directly, both by performing longer-term serial passaging and with more replicates.

Within the presented system, and more broadly in spatially organised cyanobacterial systems, it is possible that anoxygenic phototrophs, which are found to associate with cyanobacteria [17, 65], benefit from the shading effects of the peripherally positioned *F. draycotensis* and oxygen gradients arising from structure formation. Light shading effects, and penetration of wavelengths of light suitable for anoxygenic photosynthesis [81] into inner layers of photogranules and mats has been shown before [8, 62, 79, 82]. Another process we found in the presented system, and that could be confined to interiors of granules, is sulfur oxidation. Sulfur cycling is commonly reported in cyanobacterial mats [10, 11] and is also predicted in genome-based analysis of bloom communities associated with *Microcystis* species [56]. In the presented system, we were not able to consistently detect presence of a hydrogen sulfide gradient through the granules, however this could have been due to variable dynamics from diffusion processes within the granule or through balanced sulfide production and consumption.

The presented study shows how microorganisms can self-organise into a stable ecosystem, with both metabolic exchanges and spatial structure formation contributing to that stability. The presented laboratory-cultivated system will enable future studies that can test the effect of different perturbations and link these to maintenance of species interactions and stable co-existence. This spatially organised system will also allow analysing overall metabolic processes in a stable microbial ecosystem both in temporal and spatial dimensions, similar to cyanobacterial mats [8, 82], but within a controlled laboratory environment. Finally, similar “construction” of nature-derived cyanobacterial communities as done here and in recent studies [17, 65, 71], will enable study of different cyanobacteria-heterotroph interactions and the relation between media conditions, and emergence of self-organising microbial ecosystems.

## Supplementary Material

Duxbury_etal_SI_FINAL_June25_wraf126

Duxbury_etal_SupplFile1_wraf126

Duxbury_etal_SupplFile2_FINAL_wraf126

Duxbury_etal_SupplFile3_FINAL_wraf126

Duxbury_etal_SupplFile4_FINAL_wraf126

## Data Availability

The data underlying this article are available in the supplementary material (*SI*) and Supplementary files. Raw sequence data from select passages and obtained genomes are submitted to the European Nucleotide Archive and will be available under project number PRJEB89032 (ERP172084).
