# Supplementary material for: Niche formation and metabolic interactions contribute to stable diversity in a spatially structured cyanobacterial community
Source: ISME J. 2025 Jun 19;19(1):wraf126. doi: 10.1093/ismejo/wraf126 (PMC12416818; doi:10.1093/ismejo/wraf126)
Supplement: Duxbury_etal_SI_FINAL_June25_wraf126 [file duxbury_etal_si_final_june25_wraf126.pdf]

Supplementary Information (SI) for:

Niche formation and metabolic interactions contribute to stable diversity in a spatially structured cyanobacterial community

Sarah J.N. Duxbury[1,†], Sebastien Raguideau[2,†], Kelsey Cremin[1], Luke Richards[1], Matej Medvecky[3], Jerko Rosko[1], Mary Coates[1], Kieran Randall[1], Jing Chen[1], Christopher Quince[2,4,5], and Orkun S. Soyer[1,*]

Affiliations: [1]School of Life Sciences, University of Warwick, Coventry, CV4 7AL, UK. [2]Organisms and Ecosystems, Earlham Institute, Norwich, NR4 7UZ, UK, Gut Microbes and Health, [3]Bioinformatics and Digital Health Services, Research Technology Platforms, University of Warwick, Coventry, CV4 7AL, UK, [4]Quadram Institute Bioscience, Norwich Research Park, Norwich, NR4 7UQ, UK, [5]Warwick Medical School, University of Warwick, Coventry, CV4 7AL, UK.

† These authors contributed equally to this work.

***Corresponding Author:** Orkun S Soyer (o.soyer@warwick.ac.uk), School of Life Sciences, University of Warwick, Coventry, CV4 7AL, UK, + 44 (0)24 7657 4251.

This file contains Supplementary Methods and Results**,** Figures S1-S17, Tables S1-S5 and References.

Four supplementary files are provided as Excel sheets:

1: Fractions of raw sequence data mapped to MAGs across samples.

2: KO and gene presence across species for photosynthesis, Rubisco and sulfur metabolism

3: List of loci across species identified as encoding degradation enzymes targeting galactose-containing polysaccharides.

4: KO and gene presence across species for vitamin synthesis and transport.

Supplementary Methods and Results

Sample collection and culture maintenance. Freshwater samples were collected from Draycote Water Reservoir, Warwickshire, UK on 5th October 2013. Samples were initially stored in lake water at room temperature in the laboratory (approximately 21°C) under static conditions and under diel light cycle provided by a fluorescent lamp (PowerPlant Sun Mate Grow CFL Reflector with 250w Warm Lamp).

Irregular culture maintenance period. Culture vessels were sealed with gas permeable film. Samples were sub-cultured in a set of media previously described for culturing of cyanobacteria: initially, samples were cultured in a minimal medium (MM) based on description in [1], consisting of a salt solution, trace metal, and vitamin mixes (see Tables S1, S4-5). Subsequently they were transferred to BG11 [2], and finally to BG11+ media (DSMZ

media reference number 1593), which differs from BG11 only in vitamin B12 addition. Irregular sub-culturing was performed in liquid media or on agar plates, however, full records of culture cycles were not kept over this initial period (six years).

Regular sub-culturing period. A single culture lineage was maintained in BG11+, without any carbon source addition. A vitamin mix was added to BG11+ (Table S1) and the final medium used is referred to as "BG11+ vitamin mix". Serial sub-cultures were grown under continuous 12h/12h light/dark cycles with white light illumination provided by a fluorescent lamp (see above). Light intensity was measured using a PAR sensor (LI-COR Quantum Sensor (LI-190R-BNC-5) and cultures were grown under 14 – 20 μmol photons m^{-2} s^{-1}. Cultures were kept at room temperature (approximately 21°C) under static conditions and in 150 ml medical flat glass bottles. Long-term sub-culture passages (P) have been maintained over more than two years. In this study, we focus on passages between "P0" and "P6" representing a period of one year. For each sub-culture, we performed a 1 in 200 dilution by transferring 150 μl of re-suspended filamentous culture into a final volume of 30 ml of BG11+ vitamin mix (Fig. 1A). Due to the in-homogeneity of the biofilm material, sampled biomass quantity at each transfer could not be fully standardised. Passages were performed every 34-38 days, although a time period of 187 days occurred between P0 and P1. Culturing conditions for the P0 culture (the first sequenced passage) differed slightly from the other cultures in that the medium contained an alternative metal mixture (Table S4) to the trace metal mix of the BG11+ medium and was cultured in a 500 ml medical flat bottle with a culture volume of 100 ml. Similar filament bundles and granules were observed in this culture vessel as in later passage cultures. Regular sub-culture passages were continued beyond P6 following mostly the procedure described above. For P8 - 10, time intervals between transfers ranged between 28 – 63 days. Light intensity and cycle were maintained but cultures were transferred to an AlgaeTron AG 230 light incubator (Photo Systems Instruments) with light supplied via cool white LEDs. From P11 onwards, dilution factor was reduced to 1:50 every 35 days. From P11 to P15, light intensity ranged between five – eight μmol photons m^{-2} s^{-1}. Cultures were grown under fluorescent lamp illumination again from P16 onwards with light intensity varying between 10 – 37 μmol photons m^{-2} s^{-1}.

Sub-culturing in presence and absence of vitamin mix. A later sample (P19) of the regular passaging regime was sampled after 20 days of growth to create new cultures, using a 1:25 dilution in each of three replicate 250 ml flasks with 100 ml culture volume. A triplicate set of cultures was created for each of the BG11+ medium conditions (either with or without vitamin mix addition). Starting biomass was sampled from the parent culture by first re-suspending all biomass attached to the flask, transferring to a Falcon tube, and shaking vigorously with sterile three mm glass beads to disperse filamentous biofilm clumps. Cultures were incubated in the AlgaeTron AG 230 light incubator (Photo Systems Instruments) under continuous 12h/12h light/dark cycles with cool white LEDs and in-built infra-red LED lights. Light intensity ranged 10 – 14 μmol photons m^{-2} s^{-1}. Following 35 days of culture growth, new sub-cultures were prepared by sub-culturing from each flask into a new culture flask under the same medium condition (following flask swirling and pipette mixing to disperse filaments), using a 1:50 dilution for a 30 ml culture volume in a 100 ml flask. This sub-culturing protocol was repeated per set of cultures up to P10. After 49 days of growth per set of cultures, total biomass was harvested per flask, transferred into Falcon tubes, and freeze-dried using an Alpha 2-4 LD plus freeze-dryer (CHRIST, Germany). Three empty Falcon tubes were processed and freeze-dried alongside, to correct for weight deviation of the tubes during processing.

A linear mixed effects model was fitted to the freeze-dried biomass data using the *lme4* package [3] with R version 4.2.3 [4]. Treatment (with or without vitamin mix) was used as fixed factor and Passage was used as a random factor. A likelihood ratio test was used to test significance of the fixed factor.

Sub-culturing in presence of glucose. A culture aged at 65 days, from the regular community passaging regime (equivalent to P34), was used as the parent/ancestral culture to initiate replicate cultures in 30 ml BG11+ vitamin mix + 0.1% glucose in 150 ml medical flat bottles sealed with gas permeable membrane. Cultures – together with a "medium only" control - were kept under 12 h dark-light cycle (light intensity was adjusted to 19 – 26 μmol $m^{-2}s^{-1}$) under static conditions and sub-cultured every 35 - 40 days, using 1:50 dilution. A total of four sub-culturing passages were performed. At each sub-culture stage a pellet of culture material per bottle was sampled for DNA extraction with the PowerSoil Pro kit, as described for the regular community passage samples.

Community sub-culturing in the presence and absence of physical agitation to disrupt structure formation. A culture aged at 35 days from the regular community passaging regime (equivalent to P32) cultured in a wide-neck 250 ml conical flask, was used as the parent/ancestral culture to initiate replicate cultures. The culture medium (BG11+ vitamin mix) was kept consistent. Five replicate cultures were prepared from this starter culture per two culture conditions: "static" and "shaken". In addition, a medium only control flask was incubated alongside under the static condition. A culture aliquot of 25 ml was sampled from the "parent" culture and added into a 50 ml falcon tube, then biofilm fragments were dispersed by adding 21 x 3 mm glass beads and shaking vigorously by hand for 2.5 minutes. Beads were then removed by centrifugation and 1.5 ml of dispersed parent sample was added to 73.5 ml of BG11+ vitamin mix, representing a 1:50 dilution. Cultures were prepared in 250 ml conical glass flasks and were incubated in an AlgaeTron AG 230 Light Incubator (Photo Systems Instruments) with an orbital shaker fitted (Unimax 1010: Heidolph, Schwabach, Germany) for the shaking condition. Light intensity was adjusted to 19 – 26 μmol $m^{-2}s^{-1}$ (setting of 30 μE) on the top of the shaker, measured with a PAR sensor (LI-COR Quantum Sensor (LI-190R-BNC-5) & Light Meter (LI-250A)). Temperature was set to 21°C. Static condition flasks were added to the same shelf off the shaker, with equivalent light intensity as on the shaker. The shaker was set to continuous orbital shaking of 180 rpm to impede spatial structures formation. After 35 days, a fresh set of culture vessels for the subsequent passage were prepared with BG11+ vitamin mix. Flasks from the shaken condition were removed from the shaker immediately before sub-culture so that they retained their homogeneous state. Per flask, a 1:50 dilution into a fresh medium flask was made following the steps above. All cultures were added back to the incubator under their respective culture environments. This passaging step was repeated until P3 and following growth of these cultures, a pellet of culture material per flask was sampled for DNA extraction with the PowerSoil Pro kit, as described for the regular community passage samples.

Cryopreservation and revival. We tested cryopreservation and revival of a sample taken from Passage 6 of the main regularly passaged community lineage. After 28 days of growth, filaments were re-suspended via pipette mixing and one ml of culture was sampled. This aliquot was preserved in 10.0% v/v glycerol, based on previously described protocols [5, 6]. The 1 ml culture aliquot was centrifuged at 10,000 *g* for five minutes until the culture was sufficiently pelleted, followed by removal of supernatant. To the pellet, 1 ml of BG11+ vitamin mix containing 10.0% v/v glycerol was added and the pellet was re-suspended by pipetting. The sample was left for a 15-minute incubation period at low light intensity (below

5 μmol m^{-2} s^{-1}). This served as equilibration to protect the cells from cryoprotectant damage. The cryotube was then stored at -80°C. After 39 days of storage at -80°C, the cryostock was revived by thawing the top of the stock so that a 300 μl aliquot could be pipetted. To wash the cells, this aliquot was added to a microcentrifuge tube then centrifuged at 6,500 *g* for five minutes. The supernatant was discarded and fresh culture medium (BG11+ vitamin mix) without cryoprotectant was added. These centrifugations and washing steps were performed twice, and then cells were re-suspended in 300 μl of fresh BG11+ vitamin mix medium. Culture aliquot was then stored at room temperature in the dark for 24 hours. To re-grow the community culture, the 300 μl aliquot was added into 14.7 ml of BG11+ vitamin mix medium in a 50 ml Erlenmeyer flask and re-grown under the same light conditions as the original culture. A sub-culture of the revived culture was prepared after 30 days of growth, confirming longer-term culture health on re-growth.

Samples used in taxonomy and coverage analyses. *For short-read sequencing:* Cell pellets were collected from a set of eight main samples. These eight samples consisted of *(i)* five samples taken from P0 and P3-6, selected for sequencing based on availability of mature granule cultures aged at 209, 254, 218, 184, and 155 days following sub-culture, respectively; *(ii)* a sample from a culture maintained in MM (see media section above), aged at 441 days since sub-culture, and *(iii)* a sample from the cryo-revived culture of passage 6 (described above) after 44 days. This last sample was compared with a similarly aged sample taken from passage 11 (collected at 49 days). An additional four samples were collected and extracted from P1, P10, P12 and P20, at 38, 63, 29 and 49 days, respectively, following sub-culture.

For analysing the insides of larger granules, mature cultures of 60 - 65 days were used. An 18G needle attached to a 1-mL syringe was inserted into the centre of a larger granule (diameter >1 mm), and the inside portion was slowly extracted, ensuring a minimum of the cyanobacterial outer layer was collected and dispensed into a sterile Eppendorf tube. This produced less than 0.1 mL of a yellow/brown substance of a wet lumpy consistency.

For long-read sequencing: Cell pellets were collected from community samples (using same methods as above) for P0 (aged 129 days since sub-culture) (sample 1) and P7 (aged 84 days since sub-culture) (sample 2), with a minimum pellet wet weight of 100 mg. For P0, pellets were stored at -80°C prior to DNA extraction, whereas for P7, pellets were snap frozen in liquid nitrogen before being stored at -80°C.

DNA extraction and sequencing of community samples. *Shotgun sequencing.* Cell pellets were collected from above-described samples and extracted using the Qiagen PowerSoil Pro kit (Hilden, Germany, Cat. No. 47014). Biomass (suspension or biofilm) was sampled in one or one and a half ml volume for liquid culture suspension or by suction onto the end of a pipette tip for large biofilm aggregates. Tubes were centrifuged at 10,000 *g* for five minutes in a microcentrifuge (Stuart Microfuge SCF2: Bibby Scientific, Staffordshire, UK). The liquid phase was discarded. Wet weights of the pellets were recorded and adjusted to the range of 0.04 – 0.25 g (grams) by sampling additional culture aliquots if necessary. When pellets were not used immediately for DNA extractions, samples were stored in the -80°C freezer until extraction. For DNA extractions, the kit protocol was followed with the following modifications. Beads from each PowerBead tube provided in the kit were carefully transferred into clean microcentrifuge tubes. Cell pellets were re-suspended in a half ml of sterile water then added to each PowerBead tube and centrifuged at 10,000 x *g* for five minutes. The liquid phase was removed before adding beads back into the PowerBead tubes. For the bead-beating step, a Vortex Genie-2 vortexer was used (Merck, Darmstadt, Germany, Cat No. Z258423) with a 24-tube adaptor (Qiagen, Cat No. 13000-V1-24) and all samples

were vortexed for 15 minutes. For all centrifugation steps, the maximum speed of the microcentrifuge (12,300 *g*) was set for two minutes. A negative control sample was included from step one of the protocol, by adding Solution CD1 to an empty PowerBead tube. DNA was stored in 75 µl volume of Solution C6 at -80°C before sending for sequencing. DNA concentration was quantified using a Nanodrop spectrophotometer (NanoPhotometer N60, Implen, München, Germany) and a Qubit fluorimeter (ThermoFisher Scientific, Waltham, USA, Cat No. Q33226). Total amounts of raw sequence data (Gb) from shotgun NovaSeq (Illumina) paired-end 150 base pair read sequencing per sample were as follows, for samples presented in Figure 1: P0 = 10.8, P3 = 15.3, P4 = 14.4, P5 = 14.7, P6 = 15.2.

PacBio HiFi sequencing. DNA extractions were performed by the Natural Environment Research Council (NERC) Environmental Omics Facility (NEOF). In brief, high molecular weight (HMW) DNA was extracted using the Macherey-Nagel NuceloBond HMW DNA kit with liquid nitrogen grinding using mortar and pestle for more than 15 minutes. The frozen culture sample was added directly to the kit buffer in a 50 ml tube to reduce any HMW DNA degradation. Proteinase K volume was doubled, compared to kit instructions. The samples were then cleaned using AMpure PB beads with four repeated cycles. DNA was extracted in small volumes to reduce contaminants and DNA degradation. DNA quality scores were 1.2 – 1.5, measured by absorbance at 260/230nm and 1.75 – 2.0 measured by absorbance at 260/280nm. PacBio DNA libraries and sequencing were completed at the Centre for Genomics Research (CGR), from extracted genomic DNA samples. Following sample QC, low input library preparation was used for sample one (sample P0) and the ultra-low input protocol was used for sample two (sample P7). Sequencing was performed on the Sequel II SMRT Cell in Circular Consensus Sequencing (CCS) run mode and raw sequence data were delivered for downstream bioinformatics analyses.

Short-read sequence assembly and binning. Samples from Passage (P)0 and 3-6 were processed using the STRONG pipeline (available at https://github.com/chrisquince/STRONG). Firstly, samples were co-assembled through metaSPAdes, resulting in a normal assembly graph and a high-resolution assembly graph retaining strain diversity as a path in the graph. Regular assembly was then binned into metagenomically assembled genomes (MAGs). Per MAG, single copy core genes (SCGs) were used to extract the part of the high-resolution assembly graph centered around these SCGs. Number of strains as well as path in the subgraphs were estimated by reconstructing the coverage in the graph for each sample of the time series. Strain specific SCGs as well as MAG and strain coverages were generated. Normalisation was calculated by sample sequencing depth (per Gbp).

Long-read sequence assembly and binning. The two PacBio HiFi samples (described above) were assembled using hifiasm-meta [7] and the resulting unitig assembly graphs were used in the downstream analyses. ORFs were called on unitigs with Prodigal (V2.6.3) [8] with option meta, and single-copy core genes (SCGs) were annotated through RPS-BLAST (v2.9.0) [9] using the pssm formatted COG database [10], which is made available by the CDD [11] as in the STRONG pipeline [12]. However, to take into account strain diversity and non bluntification of the assembly graph, SCGs were clustered with MMseqs2 [13] (v13.45111) with options --min-seq-id 0.99 -c 0.80 --cov-mode 2 --max-seqs 10000. Reads were mapped to the assembly with minimap2 (2.17-r974-dirty) [14] using preset -ax asm10. Coverage was obtained from alignment using samtools (1.17) [15] and bedtools (v2.25.0) [16]. The unitigs were then binned from their coverage and composition, using both binning software CONCOCT [17] and metabat2 [18], respectively allowing unitigs as small as

1000bp and 1500bp. The two resulting sets of MAGs were combined in a unique set using custom scripts resulting in 18 high quality bins (greater than 75.0% completeness of SCGs in single-copy). For two high abundance species, *F. draycotensis* and the species from the *Chryseoglobus* genus- for which the genome was split over three bins- two separate complex (high variability) circular components were observed in the assembly graph. These appeared to be artifacts of high coverage as well as numerous wide overlapping regions between contigs. Strain diversity was explored as an explanation but no divergence in SCGs and 16S rRNA sequences nor strain diversity were detected. Thus, in these cases, we found circular consensus paths using maximum aggregate coverage depths. Of the resulting 16 long-read genomes (MAGs), five were contained in a single, circular contig indicating high quality assemblies, whereas 11 were split over multiple contigs (see Table S2). We taxonomically classified MAGs with GTDB-Tk v2.1.0 [19] and data version r207, using standard settings on GTDB-Tk, revealing that two of these were strains of *Allorhizobium rhizophilum*. For the MAG identified as deriving from the *Phreatobacter* genus, genome size was small and completeness based on Check M using the DFAST annotation platform [20] (see below) was only 48.4%. We improved on this by using the connected graph component for this MAG instead (Comp 243), which had higher completeness (see Table S2).

To assess the fraction of the total diversity represented by this collection of MAGs, 16S genes were annotated in the assembly using Barrnap [21] and clustered into OTUs using VSEARCH [22] with 97.0% identity. Each of the resulting 14 distinct OTUs (species and strains of the *Allorhizobium* genus were clustered together) could be mapped to one or more MAGs, which allows to ascertain that the full genomic diversity in the dataset was converted into MAGs.

Meta transcriptomics sequencing and analysis. Three independent, mature cultures of different ages (93, 111, and 160 days old) were sampled for RNA extraction. RNA was extracted using Monarch total RNA miniprep kit after 15 minutes of grinding with Qiagen powersoil pro grinding tube. TruSeq Stranded Total RNA Library Prep and Illumina Ribo-Zero Plus rRNA Depletion Kits were used for rRNA depletion. Library preparation was done using Novogene NGS RNA Library Prep Set (PT042). The library was checked with Qubit and realtime PCR for quantification and bioanalyzer for size distribution detection. Sequencing with Illumina Hiseq PE150 yielded about 10GB sequence and 67 million reads per sample, above the suggested 40-50 million reads for good quality metatranscriptomics analysis [23]. Sequences were analysed using the SAMSA2 pipeline [23] with standard settings and mapping reads to long-read generated, high-quality genomes.

Genome annotations for metabolic pathways. Genome annotations were performed using the DFAST annotation platform (releases 1.2.15/1.2.18; [20]). DFAST was run per genome with settings additional to the defaults as follows: perform taxonomy and completeness checks, use Prodigal for annotation of the coding sequence, set E-value to 1e-10 and enable both HMM scan and RPSBLAST. For the cyanobacterial genome, the Cyanobase organism-specific database was selected. Resulting protein sequences from genome annotation were further annotated for KEGG orthologs (KOs) using KEGG database [24] and KofamKOALA (release 102.0/103.0; [25]). Resulting KO lists from short and long-read data were concatenated to create a unique KO list (where possible) for each of the 17 species in the final set. KO lists were combined for the two bins predicted to represent two different strains of *Allorhizobium rhizophilum* in the long-read data. Individual KOs were manually searched for genes of interest (listed in *Supplementary files 2 and 4*) and analysed as described below. Data were plotted using R version 4.1.2 [26] and package MetQy (version 1.1.0) [27].

Analysis of monosaccharide degradation pathways and transporters. Monosaccharides described as key components of cyanobacterial slime (see main text) were searched for degradation pathways and transporters on both the KEGG [24] and MetaCyc [28], and covering those described in the dbCAN database [29, 30]. In many cases, KEGG and MetaCyc pathways were overlapping. Fig. S7 presents gene presence/absence for pathways and transporters for which at least partial presence was detected across species.

Analysis of vitamin biosynthesis pathways. Presence of vitamin biosynthesis pathways and transporters were analysed for the concatenated set of KOs and protein list combined from short and long read genomes per species. The ten vitamins present in the vitamin mix (Table S1) were analysed. Firstly, completeness of KEGG modules for biosynthesis of each vitamin were searched on the KEGG database [24] and presence of required KOs according to KEGG logic. Secondly, biosynthesis pathways and transporters for each vitamin were searched on the MetaCyc database [28]. In cases where alternative pathways (described in bacterial species) existed in MetaCyc and gene presence was complete for at least one community species, these additional pathways were also analysed / presented. The same process applied when analysing transporter presence, ensuring that all genes within multi-gene operons were analysed / presented. Genes identified as vitamin transporters in a wide environmental study [31] were also analysed. The list of detected synthesis pathways and transporters are reported in *Supplementary File 4* and overall completion is summarised in Fig. 3A.

As we have identified two key vitamins – vitamin B7 (biotin) and B12 (cobalamin) – for which biosynthetic pathway completeness greatly varied across species, analysis of these pathways is described in greater detail. Vitamin B7 (biotin) biosynthesis consists of a two-part pathway (illustrated in Fig. 3) [32] and the genes *bio ABDF* of the lower pathway were mostly absent across all species apart from *P. composti* (Fig. 3 and *Supplementary file* 4). The vitamin B12 (cobalamin) biosynthesis pathway consists of more than 30 genes, involving corrin ring synthesis and the nucleotide loop assembly [33, 34] (Fig. S11 and *Supplementary file* 4). Additionally, synthesis of the ligand DMB (5,6-dimethylbenzimidazole) from riboflavin is required [35-6]. Capability for riboflavin biosynthesis is present across several genomes (Fig. S11). Cells can alternatively transport corrinoid compounds (such as the intermediate cobinamide) into the cell and convert the intermediate cobinamide into cobalamin via a scavenging pathway [37] (Fig S11). This pathway shares overlapping genes with those of the nucleotide loop assembly. Some transporters for cobalamin import have been differentially characterised for gram-negative and positive bacteria [37, 38] (Fig S11) however the presence of these was mainly incomplete across our species. Vitamin B12 biosynthesis is so far known to be restricted to certain bacterial species [38-9] however species variation in genes encoding biosynthesis and transport pathways is under-characterised. The alternative Gram-negative transporter BtuM can substitute for BtuCDF [35] but was not present in the concatenated protein list of all species in either short or long read genomes.

Analysis of amino acid biosynthesis pathways. Completeness of KEGG modules for biosynthesis of each amino acid were searched on the KEGG database [24] and presence of required KOs according to KEGG logic were searched across the genomes using MetQy tool [27] (completeness scores, where above 0.25, are shown in Fig. S9).

Phylogenetic analyses. Taxonomic assignment of metagenomes and circularised genomes was performed using GTDB-Tk v2.1.1 [19] and data version r207, using standard settings on GTDB-Tk. Out of the 17 MAGs identified, 10 show high similarity to cultured or sequenced

genomes and were assigned by GTDB-Tk at genus level, one was assigned at family level whilst the other five were assigned at species level (see Table 1).

In the case of the cyanobacterium found in the presented community, there was high sequence similarity to only one other uncultured metagenome in the databases (GTDB id; JAAUUE01 sp012031635). This prompted us to further explore the taxonomic placement and run an additional phylogenetic analysis. To do so, we used the multiple sequence alignment of the single copy core genes (SCGs) – as created by the GTDB-Tk platform – to create an alignment of the cyanobacterium MAG identified in this study, all the GTDB species/MAGs from the *Cyanobacteriales* order, and the *Pseudomonas_E composti* MAG identified in this study (as an outgroup). We then used this alignment to build a maximum likelihood tree with FastTree [40] using the default options. The resulting tree, re-rooted at the *P. composti* MAG, is shown in Fig. S2.

Species isolation. Isolation of species from the community was achieved using three types of agar with carbon source supplementation: BG11+ vitamin mix with 0.1% w/v glucose, yeast mannitol and BG11+ vitamin mix with 0.05% w/v riboflavin. Yeast mannitol was selected to enrich for *A. rhizophilum* and was prepared following the methods of [41]. Riboflavin was chosen to select for *M. maritypicum* based on previous description of riboflavin degradation by this species [42]. Addition of glucose was expected to generally enrich for heterotrophs.

Later passage cultures were selected to attempt isolation, using pipette mixing to resuspend filaments. For BG11+ vitamin mix with 0.1% w/v glucose, a 10 µl aliquot from passage 8, aged 42 days old, was sampled and successively streaked across an agar plate. Additionally, a later passage culture was diluted into BG11+ vitamin mix with 0.1% w/v glucose medium and subsequently aliquoted and streaked onto agar plates of the same medium and LB plates. For yeast mannitol, a dilution of 1000-fold was first prepared from passage 7, aged 60 days old. A spread plate was prepared with a 50 µl aliquot from this dilution. For BG11+ vitamin mix with 0.05% w/v riboflavin, a 100 µl aliquot was sampled from passage 12 at 57 days old and a spread plate was prepared. Agar plates were either incubated under the same light conditions as the community culture (see above) for BG11+ vitamin mix + 0.1% glucose, or in the dark at 30°C for two – three days, or up to nine days for BG11+ vitamin mix with riboflavin due to slower colony growth. Single colonies observed on each agar type were re-streaked onto new agar plates of the same type either once more (riboflavin agar), three times (glucose agar) or four times (yeast mannitol agar) until maintenance of the colony morphotype was clear. Plates were incubated at 30°C in the dark. On the glucose plate, a cream-coloured smooth margin colony type was observed. On the yeast mannitol plate, round cream colonies were observed with a gelatinous texture. On the riboflavin plate, small, cream, round colonies were visible and bleaching of the plate occurred due to riboflavin breakdown [43].

A liquid culture of each colony type was prepared to harvest material for DNA sequencing and for cryopreservation. A single colony was inoculated into 30 ml of each respective medium and grown in a 100 ml Erlenmeyer flask, incubated at 30°C and 180 rpm for 48 hours. For the species isolated on BG11+ vitamin mix with 0.05% w/v riboflavin, the liquid culture was prepared in LB medium as growth was unsuccessful in liquid riboflavin medium. A liquid LB culture was also prepared for the species isolated on BG11+ vitamin mix + 0.1% glucose and LB agar plates. Cryostocks were prepared with a final concentration of 15.0% v/v glycerol for the cultures grown in BG11+ media and with 30.0% v/v glycerol (as of [44]) for the culture grown in yeast mannitol medium. Cryostocks were stored at -80°C. The remaining cultures were pelleted at 4000 rpm for 10 minutes. DNA was extracted from the cell pellet using the Qiagen PowerBiofilm kit (Cat. No. 24000-50) and stored at -

80°C. DNA concentration was quantified using the Nanodrop spectrophotometer. Isolates were characterised via Sanger sequencing of the bacterial 16S rRNA V3-V4 gene region.

Species characterisation via 16S rRNA gene region amplification. Isolates were characterised via Sanger sequencing of the bacterial 16S rRNA V3-V4 gene region using primers 341F and 806R as described in [45]. To distinguish between the two species of the *Allorhizobium* genus, a longer region was amplified using primer 341F with 1391R [46] as sequence differences occurred between the two species in this region. DNA was diluted to a concentration of approximately 10 ng/µl and added in 5 µl volume to the PCR amplification mix of 25 µl final volume. The reaction mix consisted of 12.5 µl of GoTaq G2 Green Master Mix (2X) (Promega Product Code: M7822), 1 µl of each of the forward and reverse primers (10 µM concentration) and 20 µl of sterile MilliQ water. For some PCR runs, a total volume of 50 µl was used and in these cases, the reagent volumes were doubled.

The PCR amplification was run using an Applied Biosystems Veriti Thermal Cycler (California, USA) with the following cycling conditions. Firstly, an initial denaturation of 3 minutes at 95°C was run. This was followed by 30 cycles consisting of denaturation for 30 seconds at 95°C, annealing at 49°C (50°C for 341F/1391R) for 30 seconds and elongation for 90 seconds (60 seconds for 341F/1391R) at 72°C. A final elongation step of 10 minutes (7 minutes for 341F/1391R) at 72°C was run followed by infinite hold at 4°C.

Products were run on a 1.0 % w/v agarose gel stained with GelRed dye (Biotium, product code: 41003) using 1 x TAE buffer, including a 100 bp ladder (NEB, product code: N3231S) mixed with purple loading dye. The gel was run at 100 V for at least 45 minutes. Bands were visualised with the gel imaging system U:Genius3 (Syngene, Cambridge, UK) via a blue LED transilluminator. A band of the correct size could be visualised between 400 and 500 base pairs for primer pair 341F/806R and around 1 kilo base pair for primer pair 341F/1391R. PCR products were purified using the GeneJet gel extraction kit (Thermo Scientific, K0691) with the following modifications. Centrifugations were performed at 12,300 *g* for 60 seconds. Sodium acetate was not added. Purified products were eluted in 20 – 50 µl of elution buffer then DNA concentration was quantified on the Nanodrop spectrophotometer. DNA was Sanger-sequenced (GATC Sequencing Service) in the forward direction using the same forward primer as in the PCR amplification. Taxonomic identity was characterised using sequence alignment to the annotated 16S rRNA gene sequences from the long-read shotgun metagenomics community data.

Characterisation of the 16S rRNA gene regions distinguished the following species: *P. composti*, *A. rhizophilum, A. sp900156055,* and *M. maritypicum*. These species were grown successfully on BG11+ vitamin mix with 0.1% w/v glucose, yeast mannitol, and BG11+ vitamin mix with 0.05% w/v riboflavin, respectively.

Isolated species physiological assays: Biolog carbon sources. To assess growth of the isolated species on different carbon sources, the Biolog phenotypic microassay "PM1 96 Carbon Utilisation Assay" (Biolog, Hayward, CA, USA) was used. This assay includes a range of possible carbon sources, including a set of amino acids, nucleotides, organic acids, polymers, sugars, sugar alcohols, and sugar phosphates [47]. Each species was revived from its cryostock on LB agar. Plates were incubated at 30°C for 24 hours (for *P. composti*) and for 48 hours for the other species (until sufficient colonies had grown). Each species was re-streaked onto a new LB agar plate before use in the Biolog assay. The inoculating fluid 1.0 times IF-0a to be used to suspend colonies swabbed from the agar plate, was prepared from 1.2 times IF-0a (Technopath, UK, product code: 72268) by dilution. For the Gram-positive protocol, an additive solution was added to the inoculating fluid consisting of the following components in their final concentrations: 2 mM magnesium chloride hexahydrate, 1 mM

calcium chloride dihydrate, 25 µM L-arginine HCl, 50 µM L-glutamate Na, 12.5 µM L-cystine pH 8.5, 25 µM 5'-UMP 2Na, 0.005% yeast extract, 0.005% tween-85.

Colonies were removed from the LB agar plate using a sterile swab and added to IF-0a to form a lightly turbid suspension. The transmittance (%T) at 600 nm wavelength (turbidity) was checked using a benchtop spectrophotometer (Spectronic 200E, Thermo Scientific), and culture density was adjusted until %T was approximately 42.0% or 81.0% for the gram-negative [48] and gram-positive [49] species respectively. For gram-negative species, cell suspension was then diluted in 1.0 x IF-0 to the starting inoculum of 85.0% T (+/- 3.0%), equivalent to 0.07 OD [48]. For gram-positive species, cell suspension was further diluted in inoculating fluid and additive solution to the starting inoculum of 98.0% T (+/- 2.0%), equivalent to 0.007 OD. Plate PM1 was inoculated with 100 µl per well of cell suspension, with a separate plate per species.

Growth was measured via absorbance (optical density at 600 nm wavelength) with continuous incubation in a plate reader (CLARIOstar, BMG LABTECH GmbH, Ortenberg, Germany) at 30°C for 48 hours. Measurements were taken every 15 minutes with double orbital shaking at 200 rpm for 5 minutes before each reading. Endpoint readings (48 hours) were used for data analysis, blank-corrected by the first or second optical density reading per well. Data were presented using R version 4.1.2 [26].

Isolated species physiological assays: growth in the presence or absence of vitamin mix. To assess growth in BG11+ medium with vitamin mix removal, we tested growth of isolated species *P. composti* and *A. rhizophilum.* Firstly, BG11+ medium was prepared as a base stock and supplemented with 0.8% w/v glucose to enable growth, then split into two media: one with vitamin mix added and one with water added as replacement. An aliquot of cryostock of each species was revived by streaking to single colonies on LB agar and incubating at 30°C for up to 48 hours. An overnight culture per species was prepared by picking an individual colony and inoculating into liquid LB medium for up to 24 hours at 30°C and 169 rpm. Cultures were then diluted to OD_{600nm} = 1.0 on a benchtop spectrophotometer, before washing twice and re-suspending in 0.9% w/v saline at equal volume. Each culture was split and re-suspended in each of the medium types. Culture was added in three (*P. composti*) or four (*A. rhizophilum*) replicate wells in 200 µl final volumes in a Greiner flat-bottomed non-treated 96-well plate per species (Catalog no: 655161), performing a 1:100 dilution into the respective medium for a starting density of approximately OD_{600nm} = 0.01. Growth was measured in the plate reader (CLARIOstar, BMG LABTECH GmbH, Ortenberg, Germany) following the settings described in the section above, for 48 hours. A path length correction of 1 cm was applied for 200 µl well volumes. Growth assays were performed on separate days for the two species.

For *A. rhizophilum*, growth was performed in BG11+ medium supplemented with 0.8% w/v glucose, with two additional medium conditions. For one condition, BG11+ medium was supplemented only with biotin at the same concentration as in the vitamin mix. For the other medium condition, supernatant from a grown culture of *P. composti* was mixed with BG11+ medium without vitamin supplementation (1.25 x reagents), in a 20:80 ratio. Supernatant was prepared as follows. A large culture of *P. composti* was grown from inoculum prepared for the 96-well plate assay described above. 200 µl of cell suspension adjusted to OD_{600nm} = 1.0 was inoculated in a final volume of 20 ml of BG11+ medium without vitamin mix for a 1:100 dilution and grown in a 50 ml falcon tube for 48 hours at 30°C and 169 rpm. Following growth, an OD_{600nm} of 0.819 was measured in a benchtop spectrophotometer. Culture supernatant was filter-sterilised through a 0.22 µm syringe filter unit and stored in the fridge until use.

Imaging of community sample. A one ml sample of culture (32 days after 1:50 subculture) was centrifuged at 4000 rpm for five minutes to pellet cells, which were then resuspended in PBS with 2.0% DMSO solution, containing a final concentration of 10 µM Thioflavin T (ThT). The sample was incubated for 20 minutes at room temperature. For post-stain washing, the sample was centrifuged at 4000 rpm for five minutes, and the pellet resuspended in fresh BG11+ vitamin mix medium in the same volume. 30 µl of stained sample was added to a BG11+ vitamin mix agar pad (1.5-2% agarose), settled for one-two minutes and then inverted into a cover-glass bottom dish (WillCo Wells, USA, HBST-5040). The sample was incubated for 30 minutes on the bench, before observing on an Olympus IX83 inverted fluorescence microscope with 10 times objective (Olympus UPLFLN10X2PH), excited with the CoolLED pE-300 light system. ThT was observed through a ECFP cube (U-F9001), whilst the fluorescence from the cyanobacterial photopigment was observed through a HcRed cube (U-F41043). ThT is known to enter bacterial cells depending on their membrane potential, and indicate their active, live state [50].

Imaging of cryo-sectioned granule sample. Granules were extracted from a mature community culture by a large pipette to minimise structure disruption and were firstly placed into PBS solution for 5 minutes, which removes amines from the culture media. To preserve the structure integrity of the granules with cryo-sectioning, the granules were first fixed. Fixing of samples was a multi-step process, whereby the granules were moved between different solutions, the solution composition and step incubation time was as follows: (1) 50 mM lysine in PBS for 10 minutes at room temperature, (2) 50 mM lysine and 2.5% v/v glutaraldehyde in PBS, for 20 minutes at room temperature, (3) 2.5% v/v glutaraldehyde in PBS, for 2 hours at room temperature. The granules were then gently washed in PBS for 5 minutes, repeated 3 times, to remove excess glutaraldehyde. Cryomoulds (Tissue-Tek, Product number: AGG4580) were partially filled with Optimal Cutting Temperature (OCT) compound (Agar Scientific, Product number: AGR1180) to create a thin even base layer, then placed on dry ice to freeze. Each fixed granule was placed onto the OCT layer within the mould. Working quickly, a corner of a tissue was used to remove excess moisture from around the granule, and then the mould was filled with further OCT to encase the granule fully and freeze dry. A Bright OTF7000 was used for cryo-sectioning, with the chamber and sample head temperature set to -14°C. The demoulded samples were allowed 20-30 minutes to equilibrate to the chamber temperature prior to slicing. The granules were sliced to a 50 µm thickness and collected on cover glass slides (No. 0 thickness). The slides were stored in a -80°C freezer when not being imaged.

The sample on the cover glass slide was then submerged in PBS for 5 minutes as a pre-stained wash step, before submerging into a PBS solution containing 2 µg/mL of Hoechst 33342, a DNA stain, for 20 minutes at room temperature. The slide was then washed by triplicate 1 mL washes of PBS, administered by gentle pipetting, to remove any residue dye. The sliced granules on the cover glass slide were imaged before staining, so to establish any background fluorescence, and afterwards again. All imaging was done on a Zeiss 880 confocal microscope with spectral emission detection capability. Tiled images were stitched within the Zeiss 880 imaging software, so to present the whole granule section.

Slime extraction and GC-MS characterisation. Slime extraction was performed adapting the methods of [51] and [52]. The final protocol is available via protocols.io [53]. The pellet underwent hydrolysis with sulfuric acid, and then trimethylsilylation in preparation for the GC-MS. GC-MS was performed on an Agilent 7890GC, coupled with a 5977B MSD detector. An Agilent HP-5MS with 5.0% Phenyl Methyl Silox column was used (30 m × 250 µm × 0.25 µm). The initial column temperature was set to 150°C, held for 2 minutes, and

increased at a rate of 8ºC/min to 250ºC, then maintained for 17.5 minutes. Helium was used as a carrier gas (1.2 mL/min). The front inlet temperature was 275°C, the transfer line temperature was 280°C, the MS source temperature was 230°C, and the MS quad temperature was 150°C. An injection volume of 1 µl was used, with an injection dispense speed of 6000 µl/min. The total run time was 32 minutes. Electron ionisation was used, with a MS scan range 50-750 m/z.

As part of the GC-MS work on monosaccharide composition of the slime/EPS material, the mass spectra can be examined to confirm a spectral match between the standards and the slime. Mass spectra were collected at different time points shown on the GC spectra (Fig. S4). Many of the monosaccharides have the same molecular mass and very similar structure, which when fractionated produce identical fragments. This is seen in the similarities between the mass spectra for the glucose and galactose standards (Fig. S4A and C, respectively). These fragment patterns match well to those seen in the slime, observed at the peaks which best match the retention times. Whilst the limited specificity of the MS data cannot absolutely confirm the identity of the monosaccharides, the similar fragment patterns combined with the matching peak retention time give confidence in identifying galactose and glucose in the slime.

Micron-scale probe measurement of oxygen and hydrogen sulfide gradients. Oxygen was measured using a Unisense OX-NP-710704 probe, whilst hydrogen sulfide was measured using a Unisense H2S-500-305717 probe. Probes were calibrated on the day of measurement, using kits provided by Unisense. The calibration kit for oxygen measurement was a Unisense zero-oxygen calibration kit (Product number: Calkit-O2) and for hydrogen sulfide measurement the kit used was a Unisense H2S/Sulf Sensor calibration kit (Product: Calkit-H2S). Granules were sampled across a set of different cyanobacterial cultures of different passage numbers and ages (Fig. 4 and S16). The microsensor was mounted onto a Scientifica IVM-Single Axis manipulator to control the probe movement. Cyanobacterial granules were extracted from cyanobacterial community samples (see above for culture details) and placed into a dish with a layer of BG11+ agar (1.5% agarose) and filled with fresh BG11+ media to cover the granule and the tip. The agar bed served as a method to protect the end of the tip from damage when it passed through the granule, it also helped to anchor the granule in a set position. The probe tip was then positioned at the approximate cyanobacterial granule surface and retracted back 1 mm for the start of the experiment. Following an initial 60-second rest period for stabilising the signal, the probe was then moved towards the granules in a step-like manner with typically a 200-300 µm step every 20 seconds. This approach allowed the current signal to reach a pseudo-steady state at each distance travelled. Data are only shown for oxygen measurements, as the hydrogen sulfide profiles were not consistent across granules. For Fig. 4, a culture equivalent to P23 was sampled. Cultures sampled for Fig. S16 are indicated in the legend.

Supplementary Figures

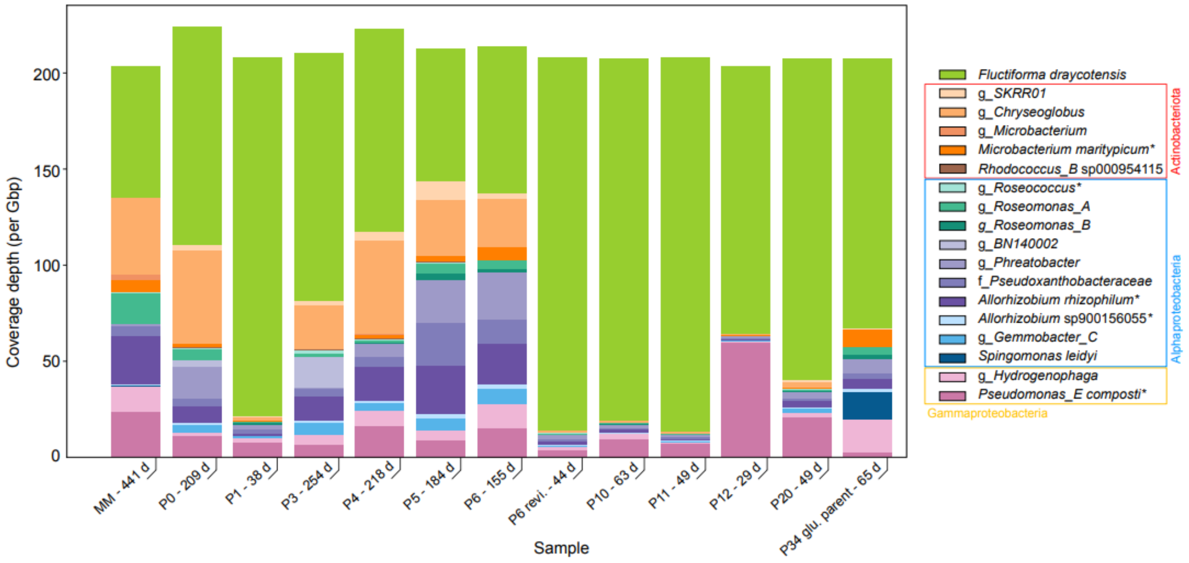

Figure S1. Coverage of taxonomic bins in additional community samples. Coverages were calculated by mapping reads from each sample, indicated on the x-axis, to the MAGs presented in Fig. 1 (see main text and above methods section for mapping details). Samples shown are indicated on the y-axis, along with age of the culture at the DNA harvest time. “MM” stands for early sample grown in minimal medium (MM) from ‘irregular passaging’ period, “P” stands for passage of regular passaging period, whereas “P6_revi” indicates a cryo-stocked sample from P6 revived. Samples P0, P3-P6 are also shown in Fig. 1 and are included here for comparison. Community composition, i.e. species coverage, colour-grouped at the taxonomic order level (listed in Table 1 and shown on the legend) as in Fig. 1, but showing additionally the *Sphingomonas leidyi* species, observed only in P34. Colour shades indicate MAGs and the highest taxonomic resolution is described in the legend on the figure. f = family; g = genus. Asterisks indicate species that were isolated from later passage community cultures. See *Methods* for sample details. The y-axis shows normalised coverage depth per Gbp of sequencing (see *Methods*).

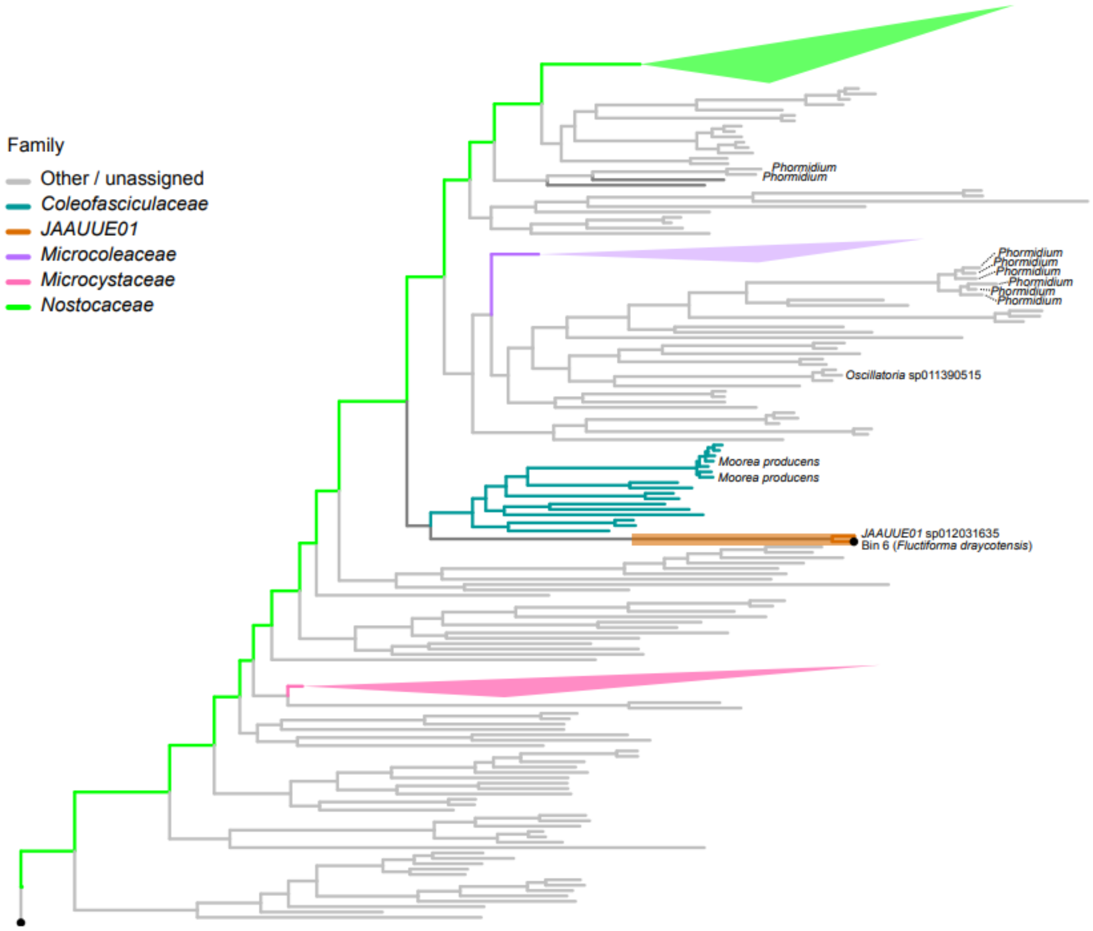

Figure S2. Maximum likelihood phylogenetic tree for the cyanobacterial species identified in this study. See *Methods* for tree creation. Species included are known species of the *Cyanobacteriales* order obtained from GTDB, the single new cyanobacterial species identified in this study (labelled as Bin 6 on the tree), and the *P. composti* bin from this study. Both of these bins are indicated with a black dot on the tree. The tree is re-rooted at the *P. composti* bin, which was included as an outgroup. The branch length to this bin was divided by 50 to better visualise the tree. Key families of the *Cyanobacteriales* order are highlighted as indicated in the legend, with the *Nostocaceae* (183 species), *Microcoleaceae* (45 species), and *Microcystaceae* (41 species) family clades collapsed to better visualise the tree. Notice that the commonly studied filamentous cyanobacterial genera *Nostoc* and *Trichodesmium* fall under the *Nostocaceae* and *Microcoleaceae* families respectively. Several species named under the *Oscillatoria* and *Phormidium* genera, which have also been studied in the past for their motility, do not form a monophyletic group. These species, as well as several species of the genus *Moorea*, studied for their natural product biosynthesis [54], are highlighted on the tree. The JAAUUE01 family is automatically generated by the GTDB database and contains only two species, the one discovered and cultured in this study, and another uncultured MAG (JAAUUE01 sp012031635) [55].

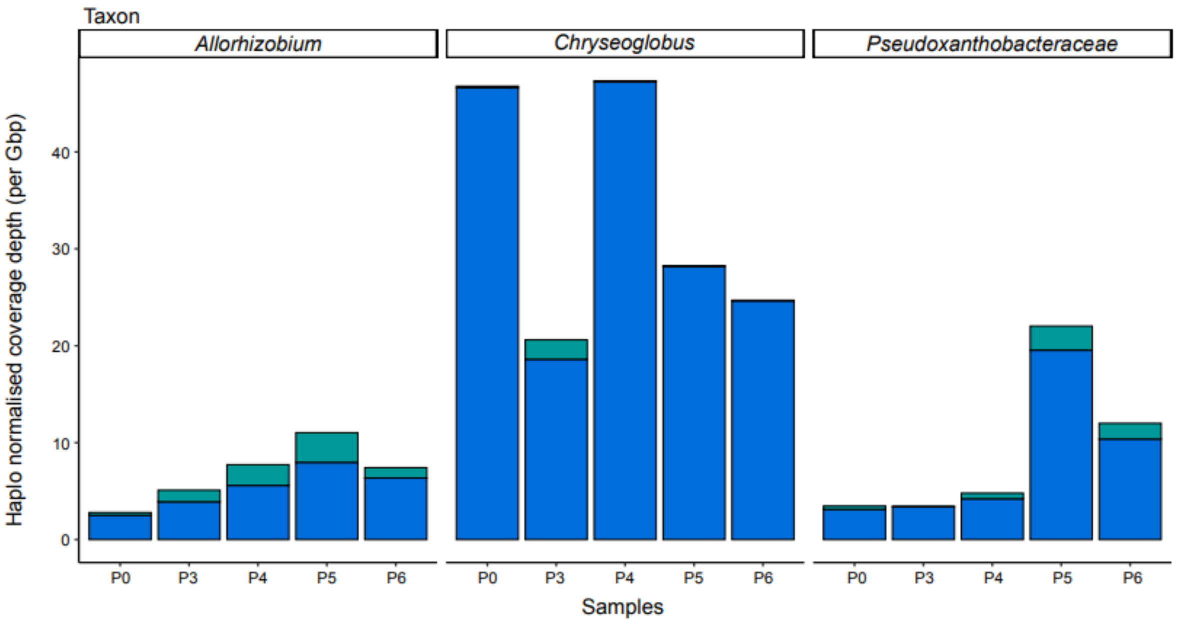

Figure S3. Within-bin haplotype coverage over passaged cultures. Coverage was calculated as an intensity value with the Bayes path algorithm, calculated as k-mer coverage across single copy genes (SCGs) divided by read length within the STRONG pipeline (see *Methods*). The intensity proportion assigned to each haplotype per bin was multiplied by the normalised coverage depth per Gbp for the bin for each passage, to calculate haplotype normalised coverage depth. Two strains were detected in each of three bins (species) indicated in the header of each sub-plot (see Table 1), over the five presented passages.

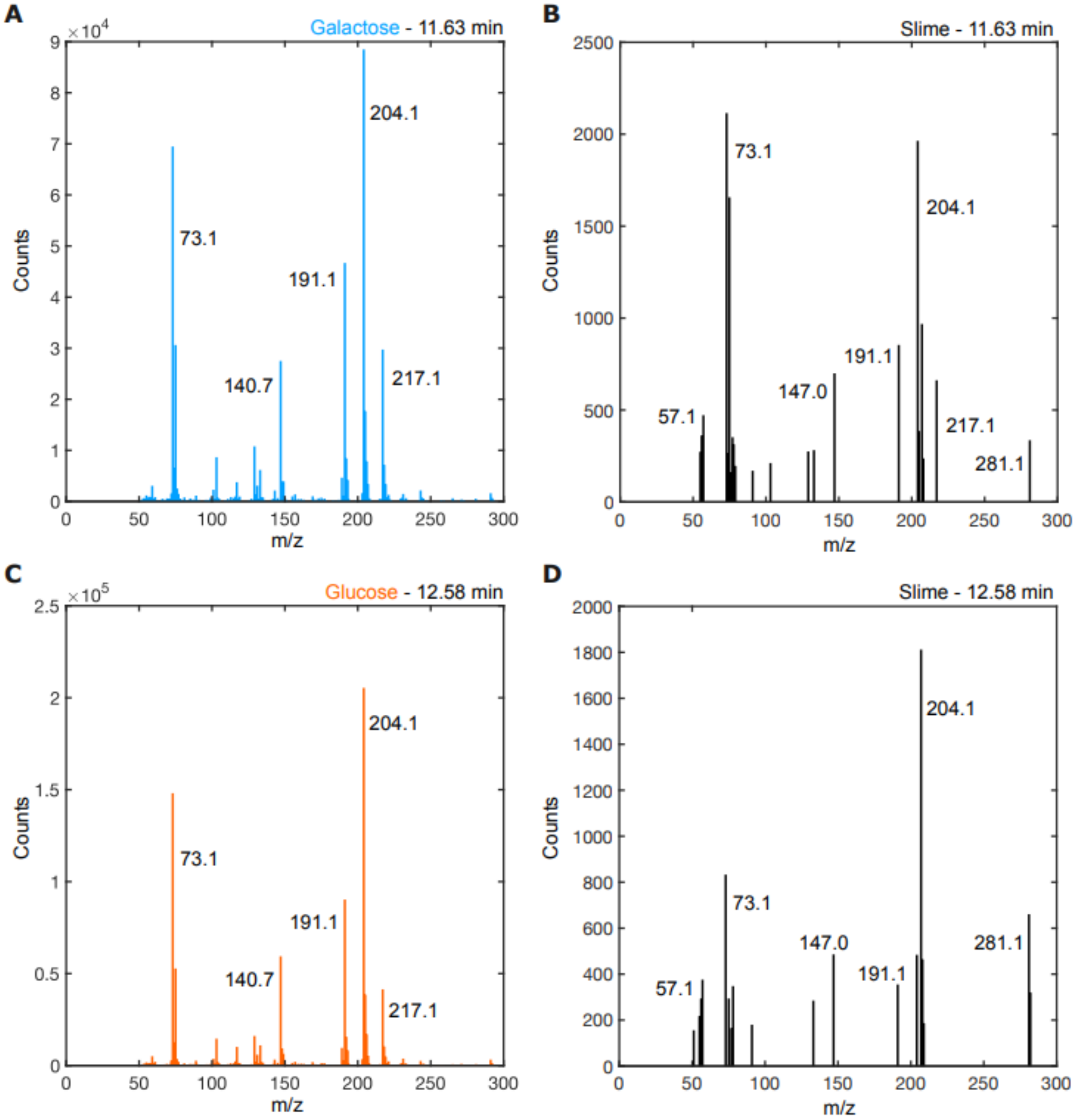

Figure S4. Accompanying mass spectra for GC analysis of cyanobacterial slime. Mass spectra at the 11.63 min peak are shown for the galactose standard **(A)** and for the spectra from the same time point in the slime GC **(B).** Mass spectra at the 12.58 min peak are shown for the glucose standard **(C)** and for the spectra from the same time in the slime GC **(D).**

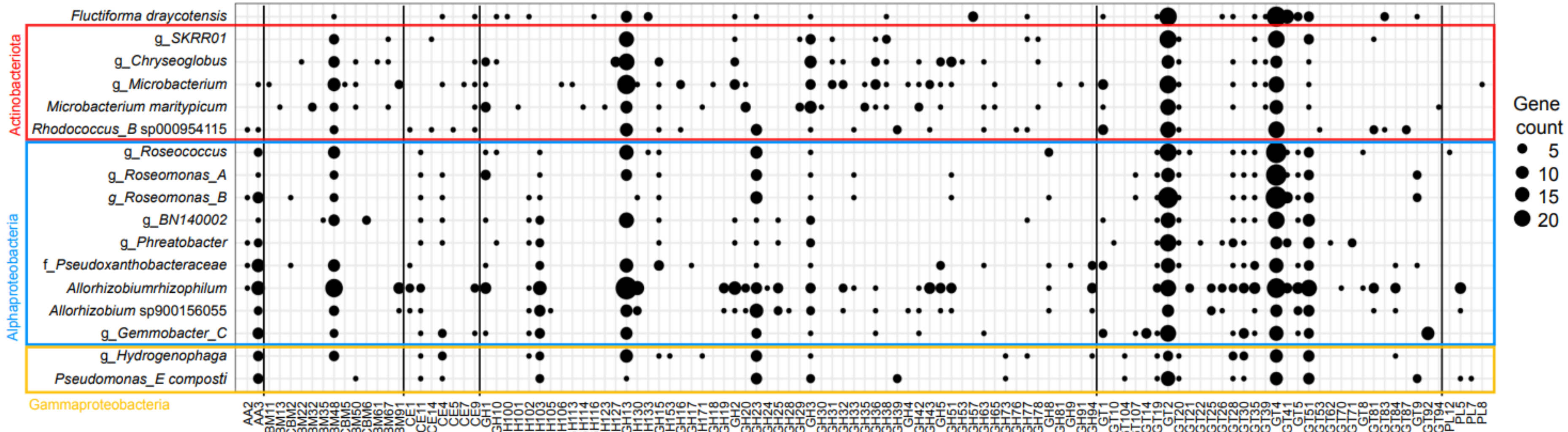

Figure S5. Carbohydrate-active enzyme (CAZy) gene families annotated in the community members' genomes. Gene count represents the number of genes detected in each species using dbCAN3 (see *SI Methods*). The group names on the x-axis correspond to the different carbohydrate active enzyme families established on the dbCAN3 database. Species are shown on the y-axis and are grouped as in Fig. 1.

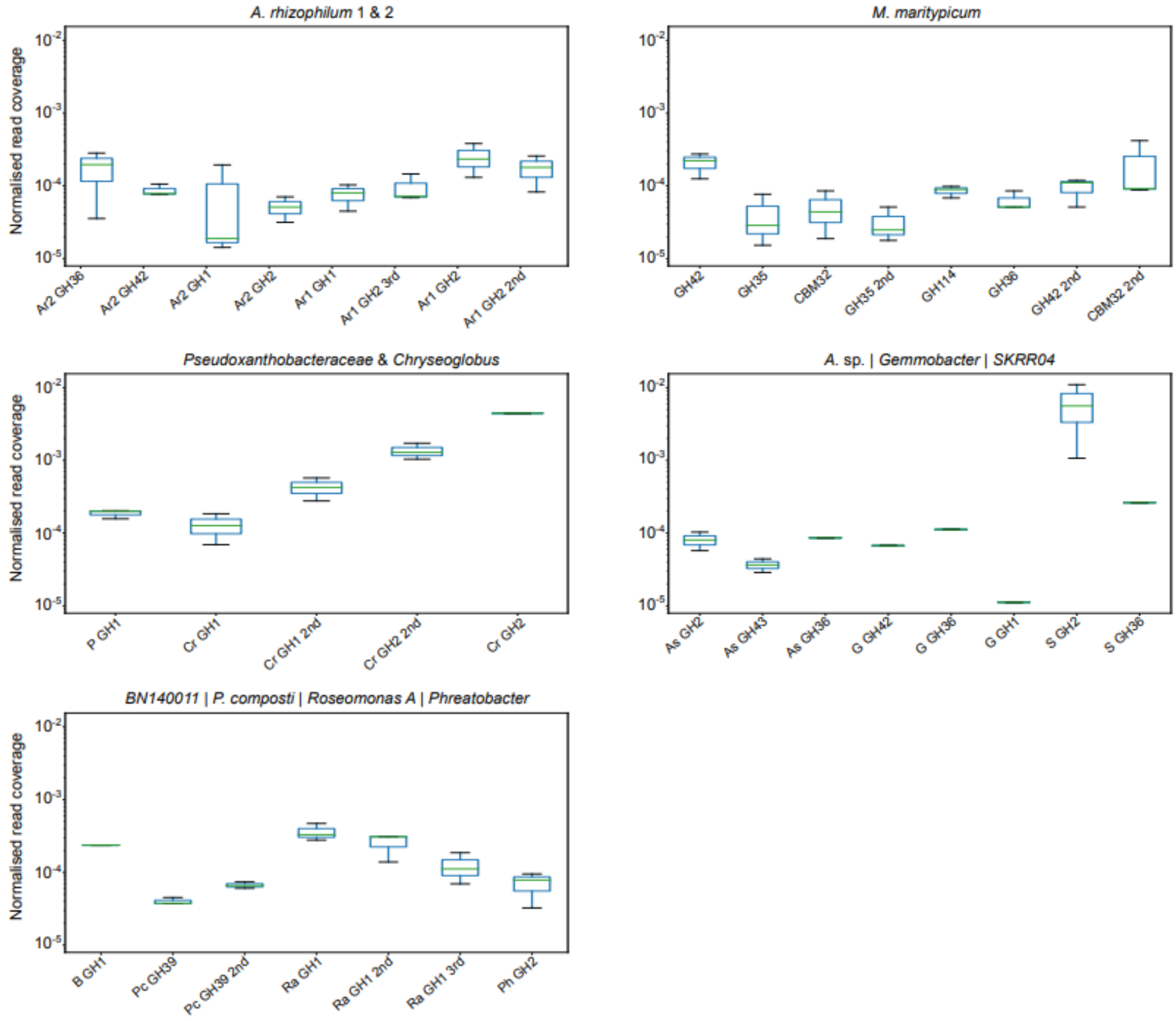

Figure S6. Meta transcriptomics based read coverage for select degradation genes targeting galactose containing carbohydrates. Expression levels of selected genes in given species, as indicated by the x-axis labels, and across three independent samples, collected from cultures aged 93, 111, or 160 days. Shown genes are those that are identified by dbCAN as belonging to a specific degradation family – see *Supplementary File* 3 for gene IDs and associated enzyme classes, as well as the full list of identified degradation genes. The label on the x-axis combines the short hand notation for a species listed in Table 1 with the dbCAN family ID that the gene belongs to. The indication of "2nd"and "3rd" are used for genes identified to be in the same dbCAN family. Each panel shows genes from a single or several species as indicated on panel title. Expression levels for each species are normalised to total expression for that species and a given sample. See details of the expression analysis in the *SI* methods.

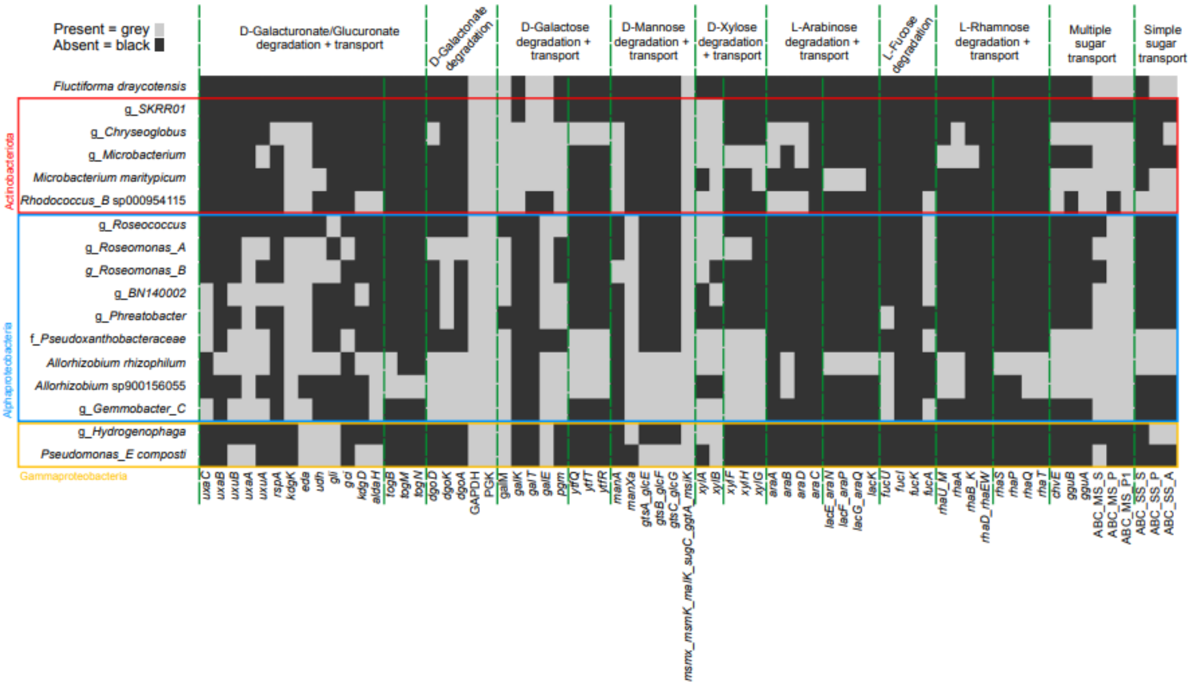

Figure S7. Monosaccharides degradation pathways and transporters identified in the community members' genomes. The presence (light grey) and absence (dark grey) of genes making up the carbohydrate degradation pathways based on KEGG and cross-checked on MetaCyc (see *SI Methods*). Gene names shown on the x-axis correspond to KOs established by KEGG or those described on MetaCyc. Genes are grouped according to the substrates and sub-divided by degradation or transport pathways they are involved in, as shown on the top of the figure and indicated with green vertical lines. Species are shown on the y-axis and are grouped as in Fig. 1.

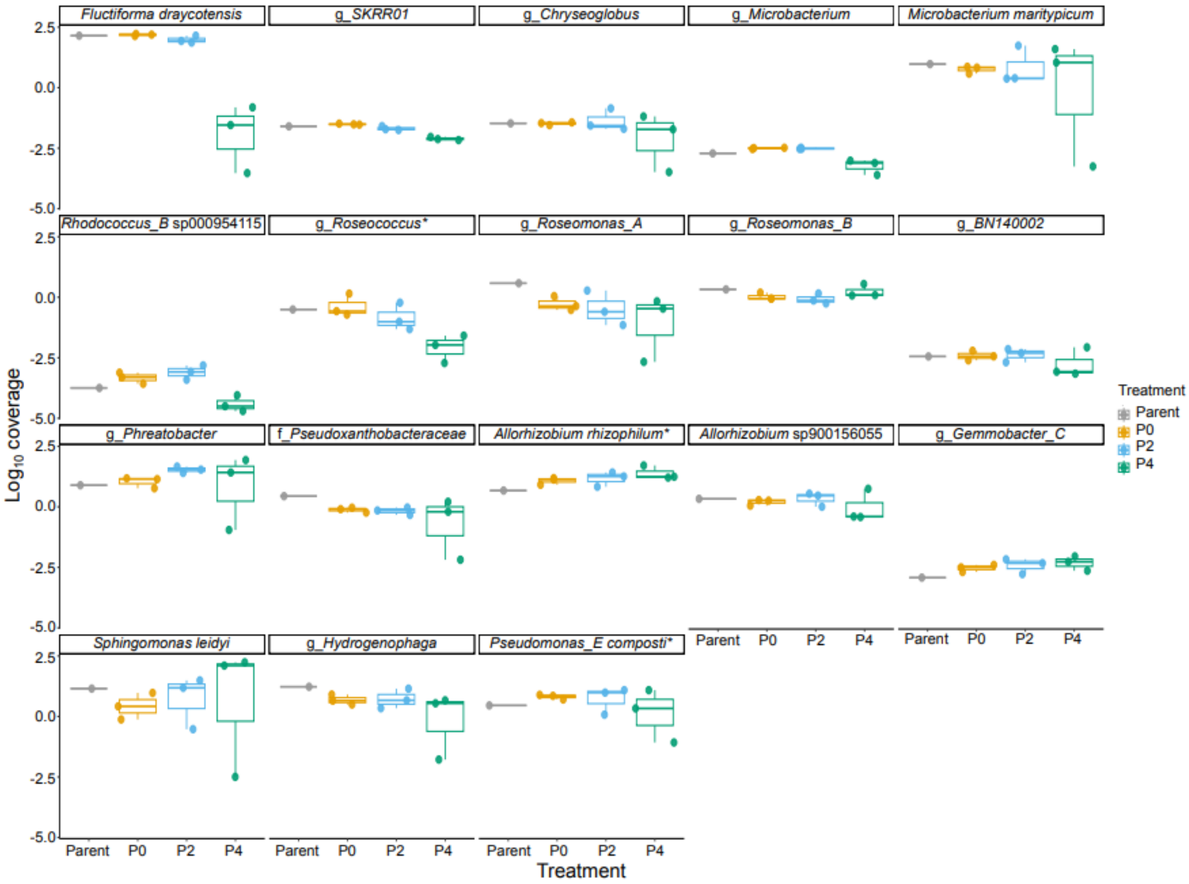

Figure S8. Coverage comparison for each species across passages in the "glucose" perturbation experiments. Each species' coverage from different passages of the experiment is shown as a box plot across three replicate cultures; P0 (orange), P2 (blue), and P4 (green). The coverage in the parent culture (grey) used to initiate the experiment is also shown. See *Methods* and the main text for further experimental details.

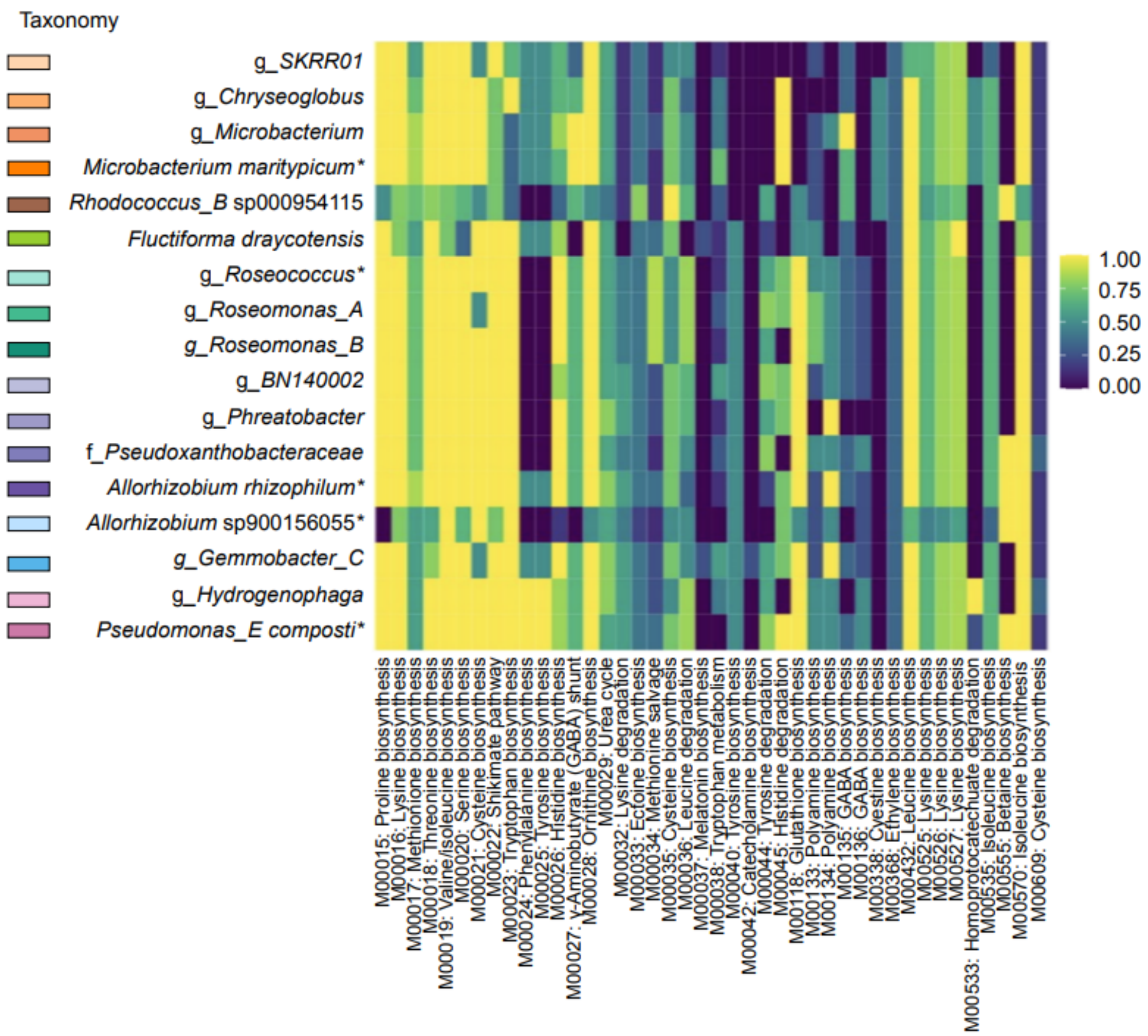

Figure S9. Completeness of KEGG metabolic modules associated with amino acid metabolism. Select modules as defined on the KEGG database [25] and analysed using the MetQy tool [24] (see *Methods*). The heatmap scale shows module completeness and only modules that were present to a level of completeness above 0.25 in at least one MAG are shown, combining annotation from short-read and long-read genomes. Bins are colour-categorised by order (as in Fig. 1) and named by the highest taxonomic resolution resolved per bin (family, genus or species). Full taxonomy is given in Table 1.

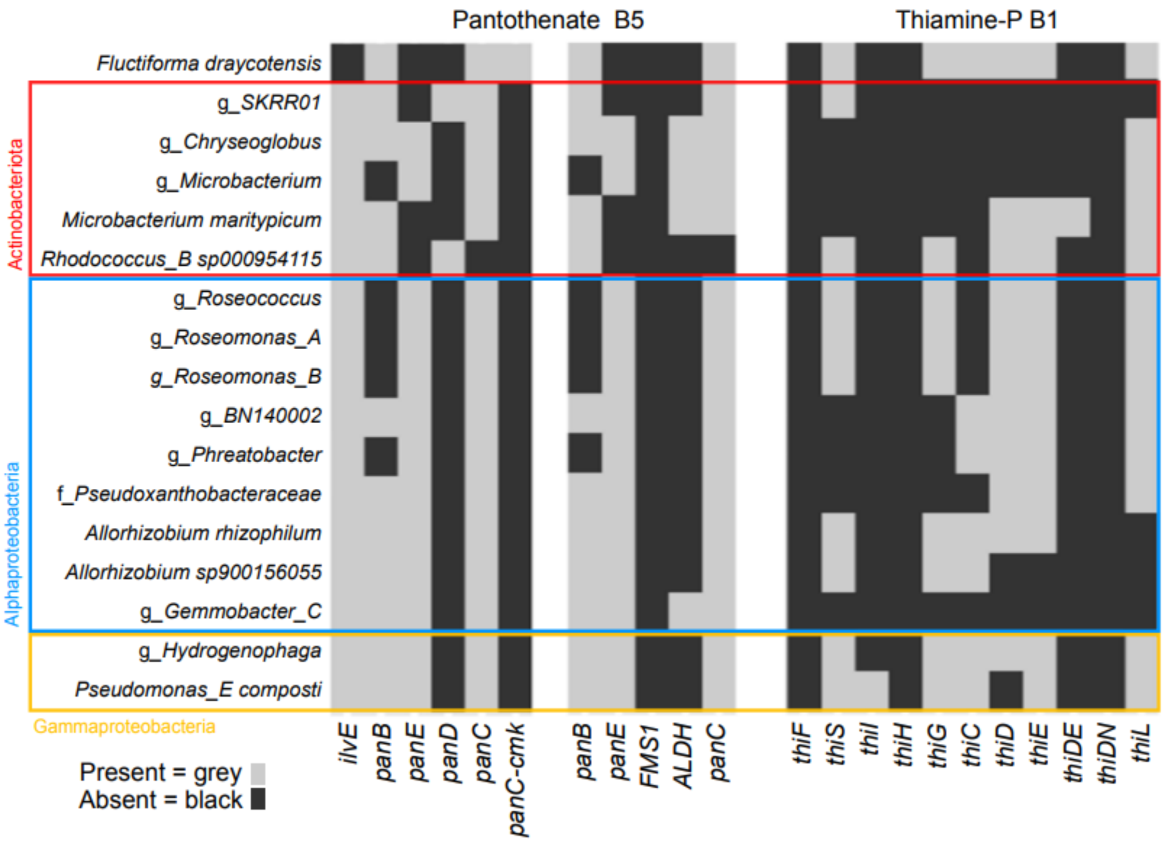

Figure S10. Analysis of vitamin B5 (pantothenate) and B1 (thiamine-P) biosynthetic pathways. Representation of completeness of pathways defined on the KEGG [25] and MetaCyc [28] databases. Heatmap plot showing presence (light grey) and absence (dark grey) of select genes across the 17 species. See Table 1 for full taxonomy. See *Methods* for full details of gene annotation and analysis, and *Supplementary file 4* for KO and gene analyses.

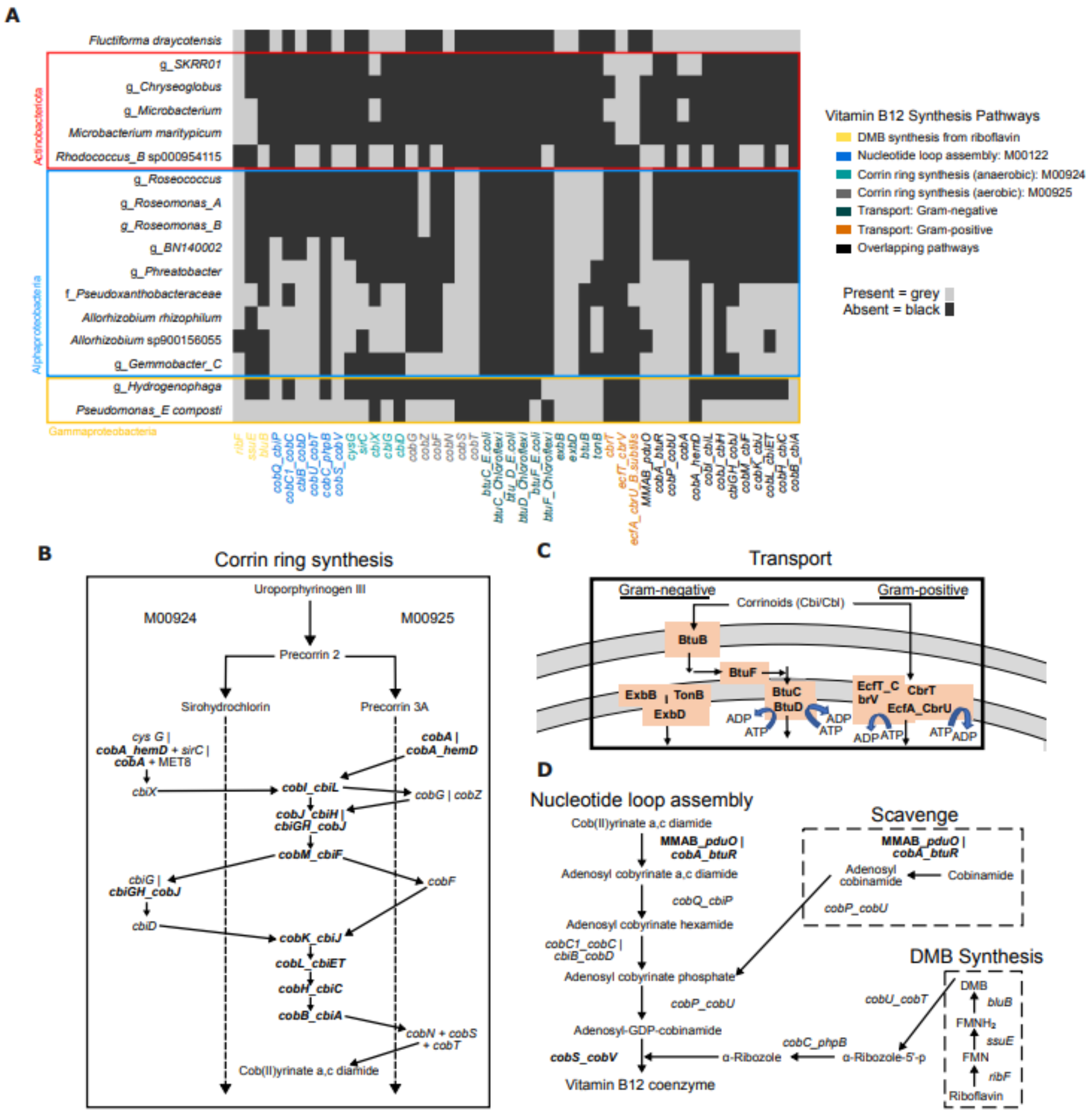

Figure S11. Analysis of vitamin B12 (cobalamin) biosynthetic and transport pathways. Representation of completeness of vitamin B12 pathways defined on the KEGG and MetaCyc databases [24, 28]. **(A)** Heatmap plot showing presence (light grey) and absence (dark grey) of select genes, colour-coded by pathway, across the final set of 17 species. See Table 1 for full taxonomy. See *Methods* for full details of gene annotation and analysis, and *Supplementary file* 4 for KO analyses. Overlapping pathway genes are those found in more than one pathway. Genes encoding the scavenging pathway directly overlap with genes required for nucleotide loop assembly **(D). (B)** Upper pathway for vitamin B12 synthesis: corrin ring synthesis, defined for both anaerobic and aerobic pathways. Genes involved in these multi-step pathways are listed separately for unique genes. Overlapping genes are highlighted in bold and those used for identical conversions are presented in the centre of the two pathways. Steps in the pathways with alternative gene options are shown with gene names separated by vertical bars. **(C)** Transport pathways of corrinoids (Cbi = cobinamide; Cbl = cobalamin) across the cell membrane(s) for Gram-negative and Gram-positive bacteria

respectively, illustrating those described in [31]. **(D)** The lower pathway of vitamin B12 synthesis as nucleotide loop assembly. This pathway is downstream of **(B).**

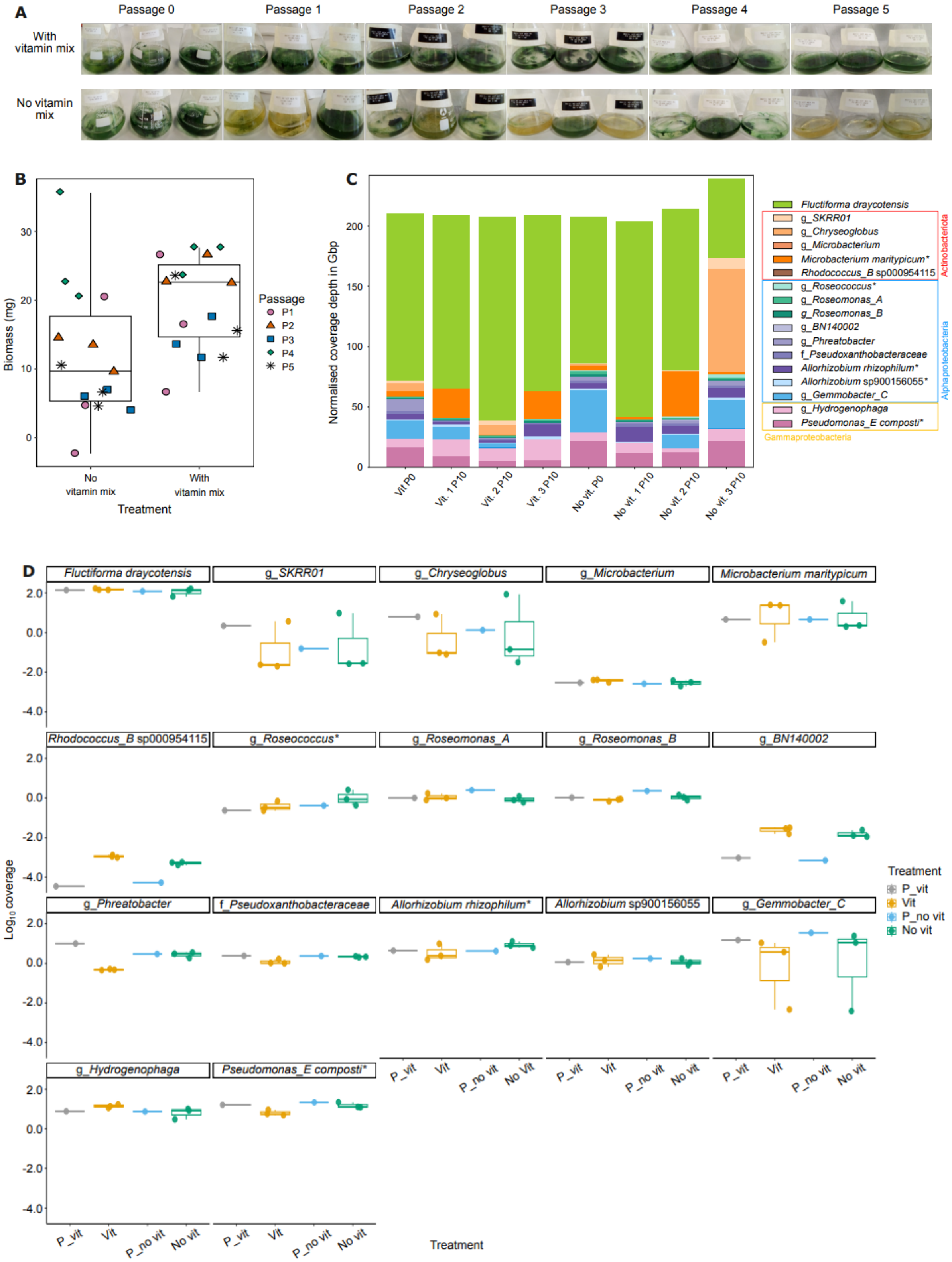

Figure S12. Growth of community cultures in BG11+ medium with and without vitamin mix supplementation. (A) Photos showing growth of three replicate cultures after 49 days growth, grown with or without vitamin mix addition, over six serial growth cycles (named Passages 0 - 5). (**B**) Total biomass weight as freeze-dried material per passage measured after 49 days of growth and shown as boxplots for the two treatments. Each treatment had three replicate cultures, which are shown as individual data points. Biomass was greater for the first passage for both treatments due to a larger culture volume, so P0 data are excluded (see *SI* and *Methods*). (**C**) Community composition (species coverage), over three replicate cultures under each treatment and assessed at the 10th sub-culture stage. Colour shades indicate MAGs (listed in Table 1 and shown on the legend) and the highest taxonomic resolution is described in the legend on the figure. f = family; g = genus. Asterisks indicate species that were experimentally isolated. Labels on the x-axis denote different samples; Vit1-3 and NoVit1-3 are the three replicate community cultures that are kept under media with and without vitamin mix respectively (see *Methods* and *SI*). The coverages are obtained by mapping metagenome reads obtained from the cultures shown on this Figure to MAGs shown in Fig. 1 (see *Methods* and *SI* for details). (**D**) Each species' coverage from the final passage of the experiment is shown as a box plot across three replicate cultures used for the "no vitamin" (green) and "vitamin" (orange) treatments, along with coverage in the corresponding parent culture used to initiate the experiment, grey and blue for vitamin and no vitamin treatments, respectively (see *Methods* and the main text for further details).

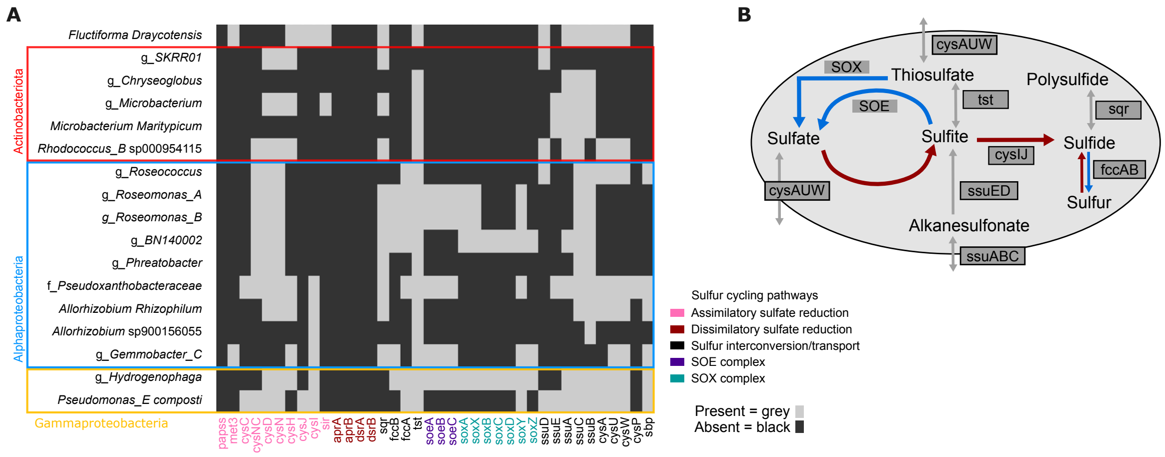

Figure S13. Functional potential for sulfur metabolism from long and short-read genomes. (A) Heatmap representation of gene presence (light grey) and absence (dark grey) for major genes encoding sulfur compound assimilation, reduction, interconversion, transport, and oxidation pathways, as defined on the KEGG database [25]. Full taxonomy is described in Table 1. Bins are colour-categorised as in Fig. 1 and named by the highest taxonomic resolution resolved per bin (genus or species). **(B)** A schematic representation of the key sulfur pathways. The oval outline represents the cell membrane, and uptake or secretion of sulfur compounds as well as their interconversions indicated with grey arrows. Blue and red coloured arrows indicate sulfur-compound oxidation and reduction respectively. Some genes analysed were absent in all genomes, particularly those encoding the

dissimilatory sulfate reduction pathway. For a full list of genes (KOs) analysed, see *Supplementary file* 2.

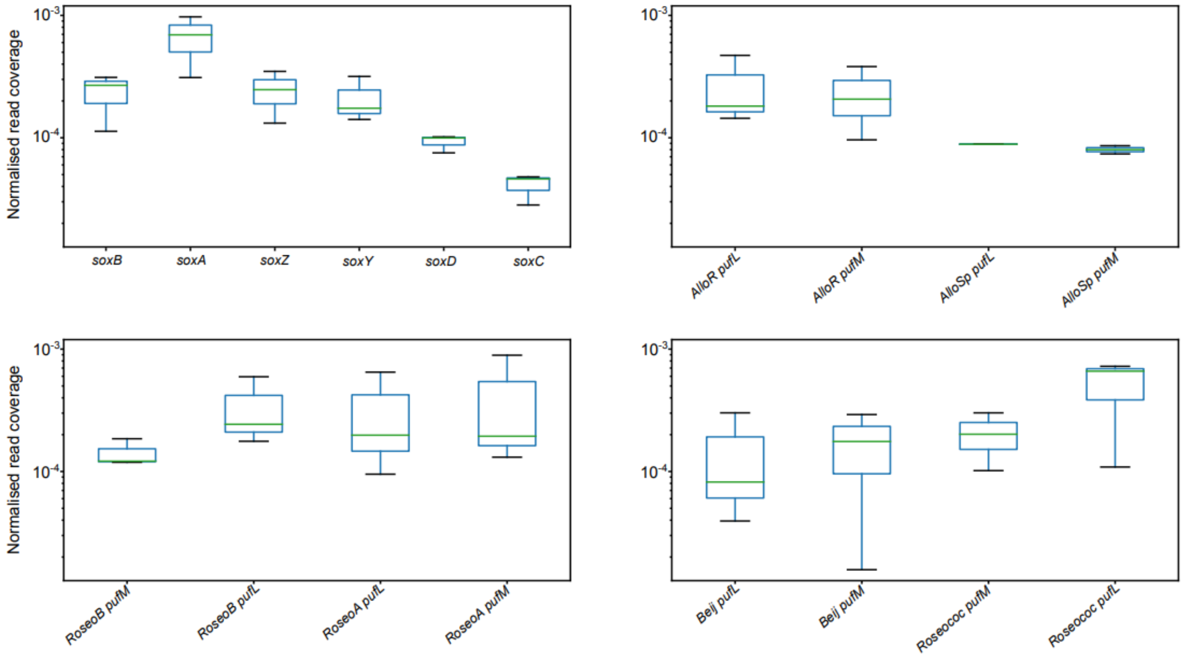

Figure S14. Meta transcriptomics based read coverage for select species and genes pairs, for genes with anoxygenic functions. Expression level of a selected gene. Top left panel shows expression levels in the species from the Hydrogenophaga genus, while for other panels, the involved species is indicated by the x-axis labels. Data is from across three independent samples collected from cultures aged 93, 111, or 160 days. Full set of gene IDs are as in *Supplementary File* 3, whereas species names are shortened version of those listed in Table 1. Expression levels for each species are normalised to total expression for that species and a given sample. See details of the expression analysis in the *SI*.

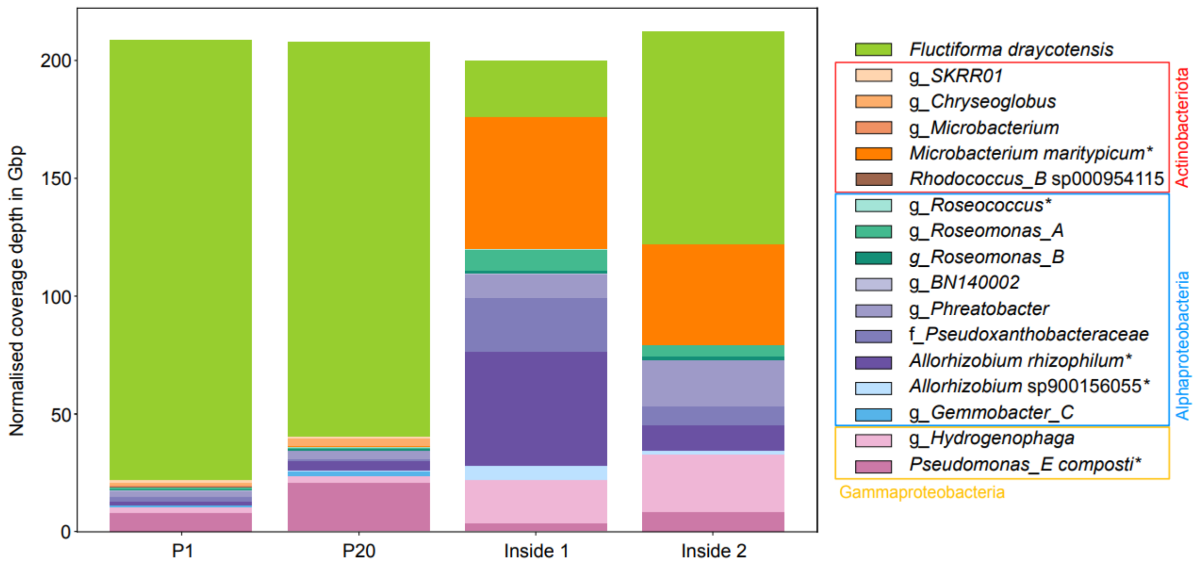

Figure S15. Coverage of bins in samples taken from inside larger granules, compared against whole-culture samples. Community composition, i.e. species coverage colour-grouped at the taxonomic order level (listed in Table 1 and shown on the legend) for the 17 species characterised. Higher taxonomic order grouping is also indicated with coloured boxes and labels on the right of the legend; f = family; g = genus. Asterisks indicate species that were experimentally isolated. Labels on the x-axis denote different samples; P1 and P20 are the same samples as shown in Fig. S1, "Inside 1" and "Inside 2" are two samples collected from the inside of large granules (see *SI*). The coverages are obtained by mapping metagenome reads obtained from the cultures shown on this Figure to MAGs shown in Fig. 1 (see main text and *SI* for details).

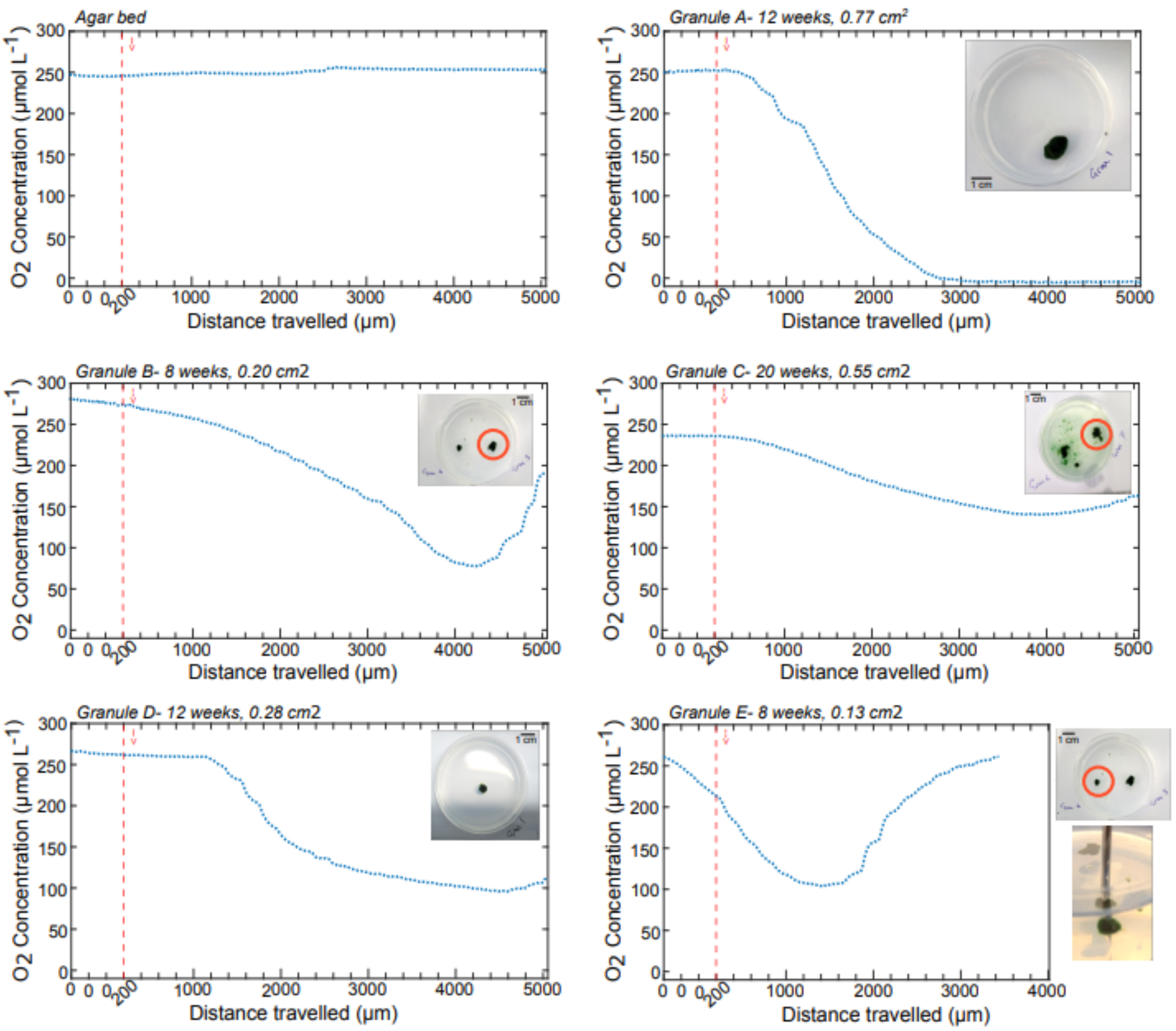

Figure S16. *Z*-axis oxygen profiles of granules. Oxygen profiles across granules of different size and age. 0 µm represents the starting position one mm above the granule, followed by the distance travelled through the granule following a timed z-axis step program (200 µm step, at each 20 sec point, unless otherwise stated). Following an initial recorded 60 second rest to allow the signal to stabilise, the red vertical line shows the first descending step of the experiment. The inset images show the investigated granule corresponding with each profile, some circled in red. The top left figure shows the oxygen profile where the experiment procedure is performed descending into the agar bed, instead of into a granule. This steady oxygen concentration measured demonstrates that the oxygen change is caused by the inside granule environment, and not from the probe moving from the BG11+ vitamin mix liquid medium into a semi-solid environment. Granules B and E show how for smaller granules, the probe can pass through the whole granule and into the agar, resulting in the oxygen concentration increasing once it has passed into the agar (as shown in the inset image where the probe has fully passed through the granule). The largest granule, Granule A, is sufficiently large that the core of the granule is fully anoxic, reaching an oxygen concentration of effectively 0 µmol l^{-1}. Granules were sampled from the following equivalent cultures: Granule A = P24, Granule B = P25, Granule C = P23, Granule D = P23, Granule E = P25.

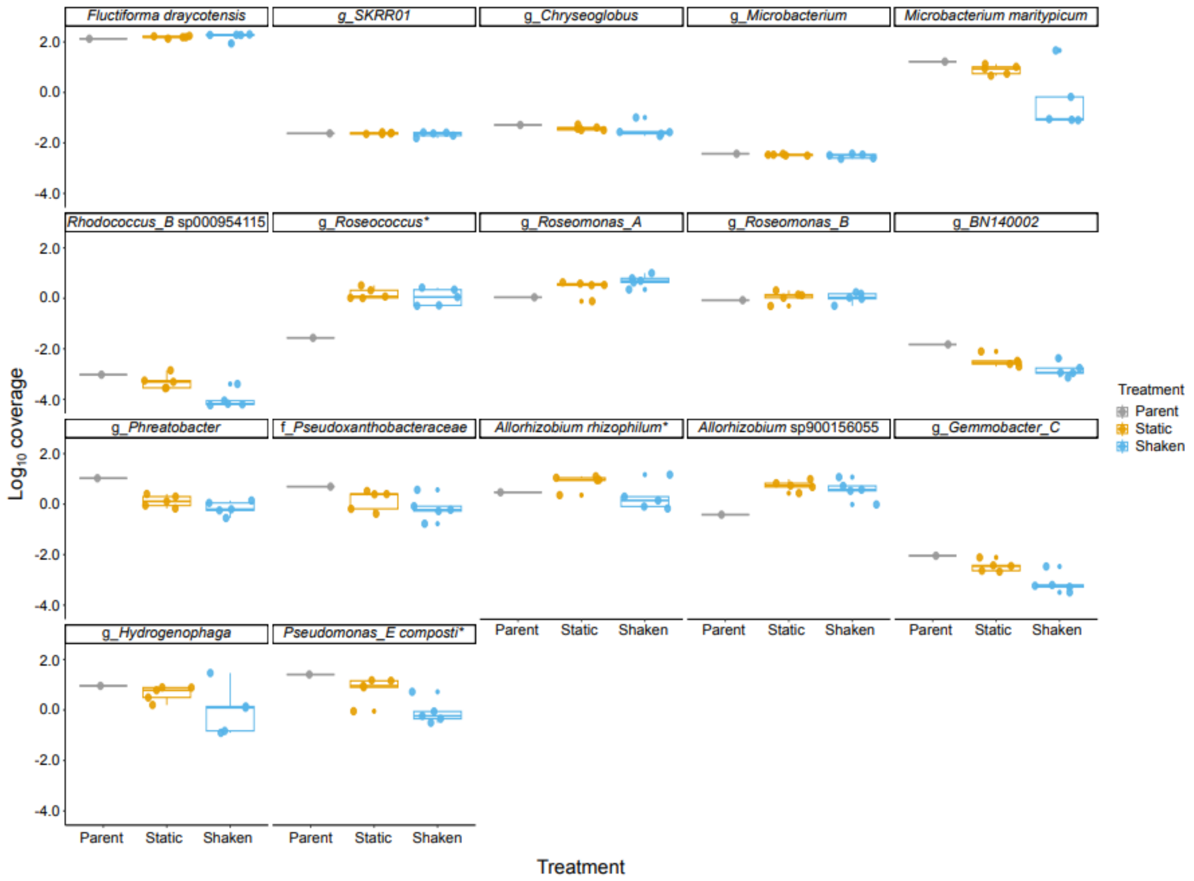

Figure S17. Coverage comparison for each species across treatments in the "shaken" perturbation experiments. Each species' coverage from the final passage of the experiment is shown as a box plot across five replicate cultures used for the shaken (blue) and static (orange) treatments, along with coverage in the parent culture (grey) used to initiate the experiment (see *Methods* and the main text).

SUPPLEMENTARY TABLES

Table S1. Vitamin mix composition for MM and for addition to BG11+ medium. This vitamin mix solution is prepared as a thousand times concentrated stock solution.

**Vitamins**	**g/l(stock)**	**g/l medium**	**g/mol**	**mol/l (medium)**
Biotin	0.020	0.000020	244.31	8.2E-08
Folic acid	0.020	0.000020	441.40	4.5E-08
Pyridoxine hydrochloride	0.100	0.000100	205.63	4.9E-07
Thiamine hydrochloride	0.050	0.000050	337.26	1.5E-07
Riboflavin	0.050	0.000050	376.36	1.3E-07
Nicotinic acid	0.050	0.000050	123.11	4.1E-07
D-Calcium pantothenate	0.050	0.000050	238.27	2.1E-07
para-Aminobenzoic acid	0.050	0.000050	137.14	3.6E-07
Cobalamin	0.001	0.000001	1355.37	7.4E-10
Lipoic acid	0.050	0.000050	206.33	2.4E-07

Table S2. Assembly and genome statistics for the 17-species bacterial community. Statistics were collected from annotation of MAGs with the DFAST pipeline [20]. PacBio long-read MAGs were analysed for all species, apart from MAGs 3 and 5 (labelled with *) for which only short-read MAGs were available. MAG completeness score represents the percentage of the total of 36 single copy core genes (SCGs) that are present, based on SCG clustering at 99.0% sequence similarity level (see *Methods*). Completeness was calculated using CheckM for MAG numbers 2, 3, 5, and 6.

MAG	Taxon	Contigs	N50 (bp)	Genome size (bp)	GC content (%)	Coding seqs	Completeness
1	g_*SKRR01*	8	4240744	4350865	71.3	4030	100.0
2	g_*Chryseoglobus*	1	2902611	2902611	65.8	2720	98.4
3 *	g_*Microbacterium*	21	279023	3753668	68.5	3489	98.0
4	s_*Microbacterium maritypicum*	1	3640567	3640567	68.4	3447	100.0
5 *	s_*Rhodococcus_B* sp000954115	1533	4110	5152295	64.9	3638	88.2
6	s_*Fluctiforma draycotensis* gen. nov., sp. nov.	1	4790552	4790552	44.3	4360	98.0
7	g_*Roseococcus*	9	4547859	4705185	71.2	4386	100.0
8	g_*Roseomonas_A*	1	6768607	6768607	70.8	6344	100.0
9	g_*Roseomonas_B*	16	3660211	5674990	71.3	5364	100.0
10	g_*BN140002*	22	412864	5357319	67.3	5265	75.0
11	g_*Phreatobacter*	13	1991051	4478198	68.8	4235	100.0
12	f_*Pseudoxanthobacteraceae*	7	4010775	5524412	70.3	5021	100.0
13_strain 1	s_*Allorhizobium rhizophilum*	75	308893	3807890	61.7	3685	80.6
13_strain 2	s_*Allorhizobium rhizophilum*	138	315789	6718987	61.6	6322	100.0
14	s_*Allorhizobium* sp900156055	92	74608	4716025	61.3	5343	88.9

15	g_*Gemmobacter_C*	41	4375673	5359230	66.6	4937	100.0
16	g_*Hydrogenophaga*	57	74981	3754005	65.6	4002	91.7
17	s_*Pseudomonas_E composti*	1	5235516	5235516	62.5	4793	100.0

Table S3. Log-10 transformed MAG normalised coverage depth per giga-base pair (Gbp) and correlations across passages. MAG coverage values normalised per Gbp of sequencing (as presented in Fig. 1) were analysed for their correlation with sample time (see *Methods* for full details). See Table 1 for taxonomy of bins. To perform Pearson correlation of each MAG with passage number, a log-10 transformation was applied to the normalised coverages. Pearson's correlation r coefficient and *P* values are presented, the latter adjusted with the Benjamini-Hochberg correction.

**MAG**	**P0**	**P3**	**P4**	**P5**	**P6**	**Pearson's Correlation r coefficient**	**Benjamini-Hochberg adjusted *P*value**
Fluctiforma draycotensis	2.053	2.110	2.023	1.838	1.881	-0.712	0.301
g_SKRR01	0.533	0.412	0.705	0.980	0.404	0.206	0.839
g_Chryseoglobus	1.682	1.348	1.686	1.462	1.404	-0.531	0.551
g_Microbacterium	-0.996	-1.106	-2.548	-2.837	-2.805	-0.875	0.220
Microbacterium maritypicum	0.359	-0.277	0.357	0.490	0.826	0.458	0.621
Rhodococcus_B _sp000954115	-0.301	-1.480	-1.480	-0.855	-3.374	-0.742	0.301
g_Roseococcus	-0.293	0.259	-0.007	-0.235	-0.373	-0.138	0.876
g_Roseomonas_A	0.722	0.278	0.008	0.754	0.625	-0.070	0.911
g_Roseomonas_B	-0.459	-0.682	-0.291	0.469	0.243	0.715	0.301
g_BN140002	0.497	1.193	-2.470	-2.358	-2.345	-0.754	0.301
g_Phreatobacter	1.231	-0.007	0.844	1.355	1.393	0.214	0.839
f_Pseudoxanthobacteraceae	0.541	0.538	0.681	1.343	1.080	0.748	0.301
Allorhizobium rhizophilum	0.943	1.100	1.250	1.400	1.327	0.941	0.220
Allorhizobium sp900156055	0.066	0.138	0.186	0.407	0.413	0.897	0.220
g_Gemmobacter_C	0.573	0.804	0.592	0.778	0.903	0.722	0.301
g_Hydrogenophaga	0.334	0.717	0.880	0.712	1.101	0.912	0.220
Pseudomonas_E composti	1.039	0.814	1.221	0.955	1.179	0.279	0.839

Table S4. Trace metals composition for MM. This trace metal solution is prepared as a hundred times concentrated stock solution.

Trace Metal	g/l(stock)	g/l medium	g/mol	mol/l (medium)
Nitrilotriacetic acid	1.5000	0.015000	191.14	7.8E-05
Magnesium chloride hexahydrate	2.4800	0.024800	203.30	1.2E-04
Manganese chloride tetrahydrate	0.5854	0.005854	197.90	3.0E-05
Sodium chloride	1.0000	0.010000	58.44	1.7E-04
Iron chloride tetrahydrate	0.0715	0.000715	198.81	3.6E-06
Cobalt chloride hexahydrate	0.1524	0.001524	237.93	6.4E-06
Calcium chloride dihydrate	0.1000	0.001000	147.01	6.8E-06
Zinc chloride tetrahydrate	0.0853	0.000853	136.32	6.3E-06
Copper chloride	0.0054	0.000054	134.45	4.0E-07
Aluminium chloride	0.0103	0.000103	133.34	7.7E-07
Boric acid	0.0100	0.000100	61.83	1.6E-06
Disodium molybdate dihydrate	0.0100	0.000100	205.92	4.9E-07
Nickel chloride hexahydrate	0.0300	0.000300	237.69	1.3E-06
Disodium selenite pentahydrate	0.0003	0.000003	262.94	1.1E-08
Sodium tungstate dihydrate	0.0080	0.000080	329.85	2.4E-07

Table S5. Salt solution composition of minimal medium (MM). This recipe is based on [1] and has been altered to include vitamin and trace metal mixtures (described below).

salt name	g/mol	g/l	mM
Potassium nitrate	101.10	5.0000	49.4544
Dipotassium phosphate	174.18	0.1145	0.6573
Magnesium sulfate	120.37	0.0200	0.1662
Iron (III) chloride hexahydrate	270.30	0.0083	0.0308
EDTA disodium salt dihydrate	372.24	0.1107	0.2974

REFERENCES

1. Murvanidze GV *et al*. Taxic responses in *Phormidium uncinatum*. *Microbiology* 1982; **128:**1623-30.
2. Stanier RY *et al*. Purification and properties of unicellular blue-green algae (order Chroococcales). *Bacteriol Rev* 1971;**35:**171-205.
3. Bates D *et al*. Fitting linear mixed-effects models using lme4. *J Stat Softw* 2015;**67:**1-48.
4. R Core Team. *R: A Language And Environment For Statistical Computing.* Vienna, Austria, 2023 (https://www.R-project.org).
5. Day JG. Cryopreservation of microalgae and cyanobacteria. *Methods Mol Biol* 2007; **368:**141-51.
6. Esteves-Ferreira AA *et al*. Comparative evaluation of different preservation methods for cyanobacterial strains. *J Appl Phycol* 2013;**25:**919-29.
7. Feng X *et al*. Metagenome assembly of high-fidelity long reads with hifiasm-meta. *Nat Methods* 2022;**19:**671-4.

8. Hyatt D *et al*. Prodigal: Prokaryotic gene recognition and translation initiation site identification. *BMC Bioinformatics* 2010;**11:**119.
9. Altschul SF *et al*. Gapped blast and psi-blast: A new generation of protein database search programs. *Nucleic Acids Res* 1997;**25:**3389-402.
10. Tatusov RL *et al*. The COG database: A tool for genome-scale analysis of protein functions and evolution. *Nucleic Acids Res* 2000;**28:**33-6.
11. Lu S *et al*. CDD/SPARCLE: The conserved domain database in 2020. *Nucleic Acids Res* 2020;**48:**D265-D8.
12. Quince C *et al*. STRONG: Metagenomics strain resolution on assembly graphs. *Genome Biol* 2021;**22:**214.
13. Steinegger M, Soding J. MMseqs2 enables sensitive protein sequence searching for the analysis of massive data sets. *Nat Biotechnol* 2017;**35:**1026-8.
14. Li H. Minimap2: Pairwise alignment for nucleotide sequences. *Bioinformatics* 2018; **34:**3094-100.
15. Li H *et al*. 1000 genome project data processing subgroup, the sequence alignment/map format and SAMtools. *Bioinformatics* 2009;**25:**2078–9.
16. Quinlan AR, Hall IM. Bedtools: A flexible suite of utilities for comparing genomic features. *Bioinformatics* 2010;**26:**841-2.
17. Alneberg J *et al*. Binning metagenomic contigs by coverage and composition. *Nat Methods* 2014;**11:**1144-6.
18. Kang DD *et al*. Metabat 2: An adaptive binning algorithm for robust and efficient genome reconstruction from metagenome assemblies. *PeerJ* 2019;**7:**e7359.
19. Chaumeil PA *et al*. GTDB-TK v2: Memory friendly classification with the genome taxonomy database. *Bioinformatics* 2022;**38:**5315-6.
20. Tanizawa Y, Fujisawa T, Nakamura Y. Dfast: A flexible prokaryotic genome annotation pipeline for faster genome publication. *Bioinformatics* 2018;**34:**1037-9.
21. Seemann T. *Barrnap 0.9 : Rapid ribosomal rna prediction.* Github, 2018 https://github.com/tseemann/barrnap. Released 28 April 2018.
22. Rognes T *et al*. Vsearch: A versatile open source tool for metagenomics. *PeerJ* 2016; **4:**e2584.
23. Westreich ST *et al*. SAMSA2: a standalone metatranscriptome analysis pipeline. *BMC Bioinformatics* 2018; **19:**175.
24. Kanehisa M *et al*. KEGG for taxonomy-based analysis of pathways and genomes. *Nucleic Acids Res* 2022;**51:**D587-D92.
25. Aramaki T *et al*. KofamKOALA: KEGG Ortholog assignment based on profile HMM and adaptive score threshold. *Bioinformatics* 2020;**36:**2251-2.
26. R Core Team. *R: A Language And Environment For Statistical Computing.* Vienna, Austria, 2021 (https://www.R-project.org).
27. Martinez-Vernon AS, Farrell F, Soyer OS. MetQy-an R package to query metabolic functions of genes and genomes. *Bioinformatics* 2018;**34:**4134-7.
28. Caspi R *et al.* The MetaCyc database of metabolic pathways and enzymes - a 2019 update. *Nucleic Acids Res* 2020;**48**:D445-D453.
29. Zheng J *et al*. dbCAN3: automated carbohydrate-active enzyme and substrate annotation, *Nucleic Acids Res*, 2023;**51**:W1
30. Yin Y *et al.* dbCAN: a web resource for automated carbohydrate-active enzyme annotation. *Nucleic Acids Res* 2012;**40**:W445–W451.
31. Rodionova I A *et al.* Genomic distribution of B-vitamin auxotrophy and uptake transporters in environmental bacteria from the Chloroflexi phylum. *Environ Microbiol Rep* 2015;**7**:204-210.

32. Lin S, Cronan JE. Closing in on complete pathways of biotin biosynthesis. *Mol Biosyst* 2011;**7:**1811-21.
33. Warren MJ *et al*. The biosynthesis of adenosylcobalamin (vitamin B12). *Nat Prod Rep* 2002;**19:**390-412.
34. Costa FG *et al*. New insights into the biosynthesis of cobamides and their use. In: H.-W. Liu, T. P. Begley, Eds. *Comprehensive Natural Products III.* Elsevier, 2020, 364-394.
35. Taga ME *et al*. BluB cannibalizes flavin to form the lower ligand of vitamin B12. *Nature* 2007;**446:**449-53.
36. Hazra AB *et al*. Anaerobic biosynthesis of the lower ligand of vitamin B12. *Proc Natl Acad Sci U S A* 2015;**112:**10792-7.
37. Santos JA *et al*. Functional and structural characterization of an ECF-type ABC transporter for vitamin B12. *Elife* 2018;**7**:e35828.
38. Fang H, Kang J, Zhang D. Microbial production of vitamin B_{12}: A review and future perspectives. *Microb Cell Fact* 2017;**16:**15.
39. Jin X *et al*. Eco-phylogenetic analyses reveal divergent evolution of vitamin B12 metabolism in the marine bacterial family 'Psychromonadaceae*'*. *Environ Microbiol Rep* 2022;**14:**147-63.
40. Price MN, Dehal PS, Arkin AP. FastTree: Computing large minimum evolution trees with profiles instead of a distance matrix. *Mol Biol Evol* 2009;**26:**1641-50.
41. Legesse SA. Isolation, identification and authentication of root nodule bacteria (Rhizobia) in promoting sustainable agricultural productivity: A review. *J Dev Soc* 2016;**6:**87-93.
42. Xu H *et al*. Identification of the first riboflavin catabolic gene cluster isolated from *Microbacterium maritypicum* G10. *J Biol Chem* 2016;**291:**23506-15.
43. Yamamoto K, Asano Y. Efficient production of lumichrome by *Microbacterium* sp. strain TPU 3598. *Appl Environ Microbiol* 2015;**81:**7360-7.
44. Lin SY *et al*. *Allorhizobium terrae* sp. nov., isolated from paddy soil, and reclassification of *Rhizobium oryziradicis* (Zhao *et al.* 2017) as *Allorhizobium oryziradicis* comb. nov. *Int J Syst Evol Microbiol* 2020;**70:**397-405.
45. Klindworth A *et al*. Evaluation of general 16S ribosomal RNA gene PCR primers for classical and next-generation sequencing-based diversity studies. *Nucleic Acids Res* 2013;**41:**e1.
46. Walker JJ, Pace NR. Phylogenetic composition of rocky mountain endolithic microbial ecosystems. *Appl Environ Microbiol* 2007;**73:**3497-504.
47. Bochner BR, Gadzinski P, Panomitros E. Phenotype microarrays for high-throughput phenotypic testing and assay of gene function. *Genome Res* 2001;**11:**1246-55.
48. Zhou L *et al*. Phenotype microarray analysis of *Escherichia coli* K-12 mutants with deletions of all two-component systems. *J Bacteriol* 2003;**185:**4956-72.
49. Guo Q *et al*. Using a phenotype microarray and transcriptome analysis to elucidate multi-drug resistance regulated by the PhoR/PhoP two-component system in *Bacillus subtilis* strain NCD-2. *Microbiol Res* 2020;**239:**126557.
50. Stratford JP *et al*. Electrically induced bacterial membrane-potential dynamics correspond to cellular proliferation capacity. *Proc Natl Acad Sci U S A* 2019;**116:**9552-7.
51. Plude JL *et al*. Chemical characterization of polysaccharide from the slime layer of the cyanobacterium *Microcystis flos-aquae* C3-40. *Appl Environ Microbiol* 1991;**57:**1696-700.
52. Nakagawa M, Takamura Y, Yagi O. Isolation and characterization of the slime from a cyanobacterium, *Microcystis aeruginosa* K-3a. *Agric Biol Chem* 1987;**51:**329-37.

53. Cremin K *et al.,* Extraction of Cyanobacterial Slime from Community Samples and Subsequent Analysis via GC-MS 2023; Protocols.io. https://www.protocols.io/view/extraction-of-cyanobacterial-slime-from-community-5qpvorw8dv4o/v1 (accessed 31 January 2024).
54. Engene N *et al*. *Moorea producens* gen. nov., sp. nov. and *Moorea bouillonii* comb. nov., tropical marine cyanobacteria rich in bioactive secondary metabolites. *Int J Syst Evol Microbiol* 2012;**62:**1171-8.
55. Waterworth SC *et al*. Conserved bacterial genomes from two geographically isolated peritidal stromatolite formations shed light on potential functional guilds. *Environ Microbiol Rep* 2021;**13:**126-37.
